# Determination of biogeochemical properties in sea waters using the inversion of the three-stream irradiance model

**DOI:** 10.1038/s41598-024-71457-5

**Published:** 2024-09-27

**Authors:** Paolo Lazzari, Mirna Gharbi Dit Kacem, Eva Álvarez, Ilya Chernov, Vincenzo Vellucci

**Affiliations:** 1 National Institute of Oceanography and Applied Geophysics-OGS, 34014 Trieste, Italy; 2 Dipartimento di Matematica e Geoscienze, Università degli Studi di Trieste, Via Valerio 12, 34127 Trieste, Italy; 3 Institute of Applied Mathematical Research, Karelian Research Centre of the Russian Academy of Sciences, Petrozavodsk, 185910 Russia; 4 Sorbonne Université, CNRS, Institut de la Mer de Villefranche, IMEV, 06230 Villefranche-sur-Mer, France; 5 Sorbonne Université, CNRS, OSU Station Marines, STAMAR, 75006 Paris, France

**Keywords:** Carbon cycle, Biogeochemistry, Environmental sciences, Ocean sciences, Marine biology

## Abstract

Inversion models, in the context of oceanography, relate the observed ocean color to the concentrations of the different biogeochemical components present in the water of the ocean. However, building accurate inversion models can be quite complex due to the many factors that can influence the observed ocean color, such as variations in the composition or the optical properties of biogeochemical products. Here we assess the feasibility of the inversion approach, by implementing the three-stream light inversion model in a one-dimensional water column configuration, represented at the BOUSSOLE site in the northwestern Mediterranean Sea. Moreover, we provide a comprehensive sensitivity analysis of the model’s skill by perturbing the parameters of the bio-optical properties and phytoplankton physiology. Analysis of the inversion indicates that the model is able to reconstruct the variability of the optical constituents. Results indicate that chlorophyll-a and coloured dissolved organic matter play a major role in light modulation. The sensitivity analysis shows that the parameterization of the ratio of chlorophyll-a to carbon is important for the performance of the inversion model. A coherent inversion model, as presented, can be used as an observational operator to assimilate remote sensing reflectance.

## Introduction

Satellites sensors provide useful data at different temporal and spatial scales to reconstruct the variability of marine ecosystems. Ocean color sensors in Earth orbit allow the derivation of spectral water leaving radiance and inference of physical and biogeochemical properties of the water masses^1^. In simple terms, the color of the sea is related to dissolved and particulate matter present in the water that for most of the ocean are of biological origin. In recent years, suitable algorithms have been developed to estimate the biogeochemical state of the oceans from measured radiance^2^. In particular, the derivation of water biogeochemical properties can be formulated in terms of the inversion of a forward problem. Mathematically, the so-called forward description resolves light propagation according to the properties of the medium in which the light propagates; the corresponding mathematical framework is well established^3^. In parallel, the inversion algorithms use the available information about the light field to retrieve the Inherent Optical Properties (IOPs) of the medium in which the light propagates. The major optical constituents of seawater in open oceans are phytoplankton, chromophoric dissolved organic matter (CDOM), and non algal particles (NAP) such as organic and mineral particles, bacteria, viruses and air bubbles. These biogeochemical components are important indicators of the ecosystem trophic regime and carbon pool formation^4^ and are among the most common products derived from inversion algorithms^2^. In addition, these indicators are extremely useful to validate surface dynamics of coupled hydrodynamic biogeochemical ocean models, by comparing the distribution of satellite-derived biogeochemical products with corresponding distributions derived from model simulations^5^.

Traditionally, biogeochemical models used a simple Beer-Lambert formulation to describe photosynthetically available radiation (PAR) propagation along the water column, i.e. the downwelling irradiance integrated over visible wavelengths (from 400  to 700 nm) that decays exponentially due to attenuation. In recent years, a number of biogeochemical models have begun to resolve spectral light propagation, to simulate photon scattering and backscattering, and to simulate the remote sensing reflectance ($R_{\rm{rs}}$)^4,6–8^. This improvement in modelling algorithms is consistent with the progressive increase in data streams received from multispectral (Global Monitoring for Environment and Security-GMES-Sentinel-3^9^) and hyperspectral (PRecursore IperSpettrale della Missione Applicativa-PRISMA-^10^; Plankton, Aerosol, Clouds, Ocean Ecosystem-PACE-^11^) satellite ocean color sensors. In this work, we aim to present an inversion approach that is fully compatible with the forward model introduced in recent multispectral marine biogeochemical models^4,6,12^. The inversion module can be used for direct assimilation of optical/radiometric measurements, as it appears to be more robust than those based on phytoplankton proxies, i.e. chlorophyll-a concentration (Chl-a), thanks to a more accurate knowledge of uncertainties in optical measurements^13^. The so-called observation operator maps measured data into model state variables, allowing correction of model trajectories and model parameters. The three-stream inversion model can be used as an observation operator to assimilate remote sensing reflectance in a model equipped with the forward three-stream model to resolve the biogeochemistry.

Typically, the approximations used in inversion algorithms to estimate data for model validation and assimilation are not the same as those used in the forward models used to solve biogeochemistry. Here we demonstrate the feasibility of the inversion approach for identifying important physical and biological processes needed to coherently map information between optical and biogeochemical model variables. The inversion study is being conducted at the BOUSSOLE site in the northwestern Mediterranean Sea^14^. Given the high availability of bio-optical data, this location is ideal for the test and skill analysis of the proposed approach.

## Results

To test the possibility and efficacy of inverting the three-stream model, we performed a series of simulations in which the input data to minimize the model error, expressed by the functional J in Eq. 1, are satellite-based remote sensing reflectances at 5 selected wavelengths (412.5, 442.5, 490, 510 and 555 nm). In the present work, they are quality checked and processed for the study area as described in the “Methods” section.

**Fig. 1:**
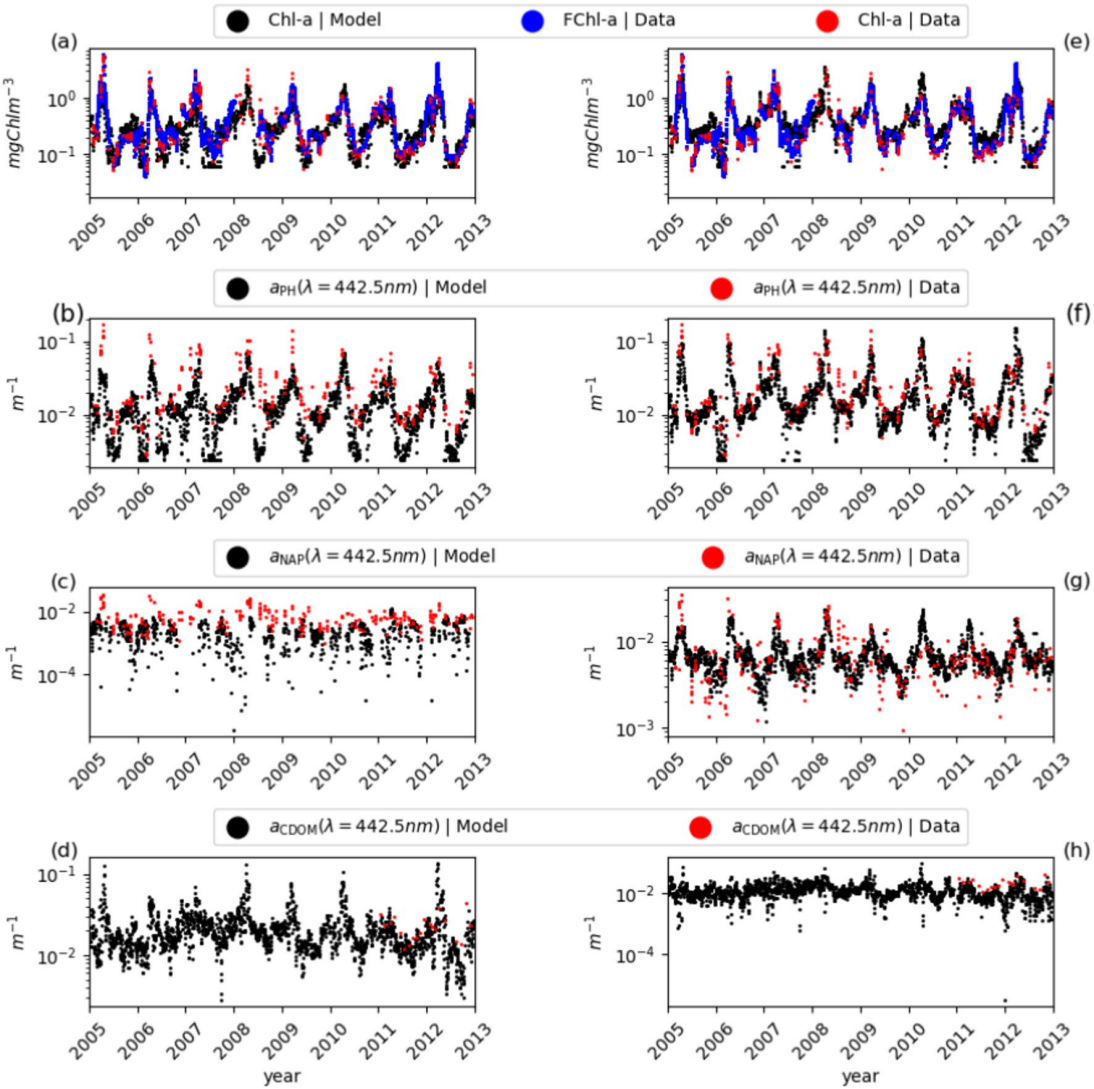
Inversion results for the years 2005 to 2012 at the BOUSSOLE site. (**a-d**) Refer to the REF model configuration and (**e–h**) to the EXP-1-Chl-a configuration, which provides the best skill metrics for Chl-a. (**a,e**) Time series of model-derived Chl-a (daily frequency, black dots), fluorescence FChl-a (daily frequency, blue dots) and HPLC measurements (monthly frequency red dots). (**b,f**) Time series of modelled and in-situ $a_{\mathrm{PH}}(442.5)$, with daily (black) and monthly frequency (red). (**c,g**) Seasonal variations of modelled and in-situ $a_{\rm{NAP}}(442.5)$ at daily (black) and monthly frequency (red). (**d,h**) Time series of modelled and in-situ $a_{\rm{CDOM}}(442.5)$ with daily (black) and monthly frequency (red). CDOM data are only available from 2011 onwards.

Based on $R_{\rm{rs}}$ information, the model provides estimates of the temporal variability of surface Chl-a and IOPs associated with phytoplankton, CDOM and NAP. We started with a reference configuration (REF) with the parameters derived from literature data and described in the Methods section. The model results of the REF output configuration show that the inversion model is able to reproduce a seasonal variability with higher Chl-a values during the spring phytoplankton bloom and lower values during the summer stratification, Fig. 1. However, the REF configuration tends to underestimate Chl-a compared to *in-situ* Chl-a for stratified periods. Phytoplankton absorption also appears to be underestimated in summer. To better understand the model results, we conducted additional experiments to test the sensitivity of the model to perturbations of the bio-optical parameters and the physiological properties of the phytoplankton. Taking into account the results of the sensitivity analysis experiments, we have defined an optimized configuration that provides the best results in terms of Chl-a (EXP-1-Chla, described in Optical configurations). In this section we analyse the results of the EXP-1-Chla configuration in comparison to the REF configuration and to the in-situ data. In both cases, thanks to the minimization of the functional J, the agreement between satellite and model $R_{\rm{rs}}$ is very high for each wavelength (see Supplementary Information).

Within the time window under consideration (2005–2012), the BOUSSOLE site exhibited Chl-a values in two orders of magnitude, ranging from oligotrophic conditions in summer (0.1 mg Chla m$^{-3}$) to mesotrophic conditions in spring (2 mg Chla m$^{-3}$). This variability is consistently reproduced by the inverse model in both the REF and EXP-1-Chl-a configurations, Fig. 1a, e. In winter, Chl-a is modulated by strong mixing events, with the phytoplankton bloom persisting on the monthly time scale. In late fall, surface Chl-a increases consistently both in observed and modeled data. The variability of Chl-a appears better reconstructed by the EXP-1-Chl-a model configuration, Fig. 1e, especially for the summer period. The performance of the two model configurations is evaluated against in-situ data and summarized in Table 1. The comparison is performed using daily model data compared with filtered daily in situ data as described in the Methods section. The Chl-a statistics show that the model tends to underestimate the annual average, while the EXP-1-Chl-a shows a positive BIAS. In the case of the RMSE, the EXP-1-Chl-a configuration shows a reduction in terms of REF. The EXP-1-Chl-a model configuration better represents the phytoplankton variability, as shown by the higher CORR value for Chl-a. Both the REF and EXP-1-Chl-a configurations are able to reproduce the interannual variability and show, for example, a lower chlorophyll concentration during the mixing period in 2011.

**Table 1:** Statistics for the period 2005 to 2012 for REF and EXP-1 ( EXP-1-Chl-a) inversion experiments.

Variable	NDATA	BIAS-REF	BIAS-EXP-1	RMSE-REF	RMSE-EXP-1	Corr-REF	Corr-EXP-1
chl-a	2607	$-$0.11	0.19	0.66	0.56	0.69	0.79
$kd_{412.5}$	792	$-$0.19	$-$0.15	0.41	0.4	0.78	0.77
$kd_{442.5}$	1824	$-$0.18	$-$0.09	0.41	0.39	0.8	0.79
$kd_{490}$	1865	$-$0.12	$-$0.04	0.39	0.37	0.77	0.76
$kd_{510}$	1820	$-$0.14	$-$0.08	0.34	0.31	0.74	0.72
$kd_{555}$	916	0.0	0.04	0.18	0.18	0.66	0.61
$bb_{442}$	1178	$-$0.83	$-$0.7	0.88	0.75	0.66	0.71
$bb_{490}$	567	$-$0.35	$-$0.28	0.54	0.49	0.5	0.55
$bb_{555}$	1158	$-$0.44	$-$0.36	0.56	0.5	0.65	0.66

The data and model results are logarithmically transformed before the statistical indicators are calculated.

The simulated phytoplankton absorption coefficient at 442 nm, Fig. 1b,f, shows similar variability to the available data and predicts higher absorption in spring and minimum values in summer, which increase further in fall. Also in this case, the EXP-1-Chl-a configuration provides a better agreement with the observed data, especially in the summer months of 2008 and 2009, when the REF underestimates the absorption. The absorption coefficient of NAP at 442.5 nm , Fig. 1c, g, shows a seasonality synchronized with Chl-a; model and data cover a similar range in the case of the EXP-1-Chl-a configuration, while it is underestimated for REF. The CDOM absorption coefficient at 442.5 nm also shows a similar seasonal variability to that observed for Chl-a. The maximum values of $a_{\rm{CDOM}}(442.5)$ between April and June coincide with the observed minimum values of Chl-a.

**Fig. 2:**
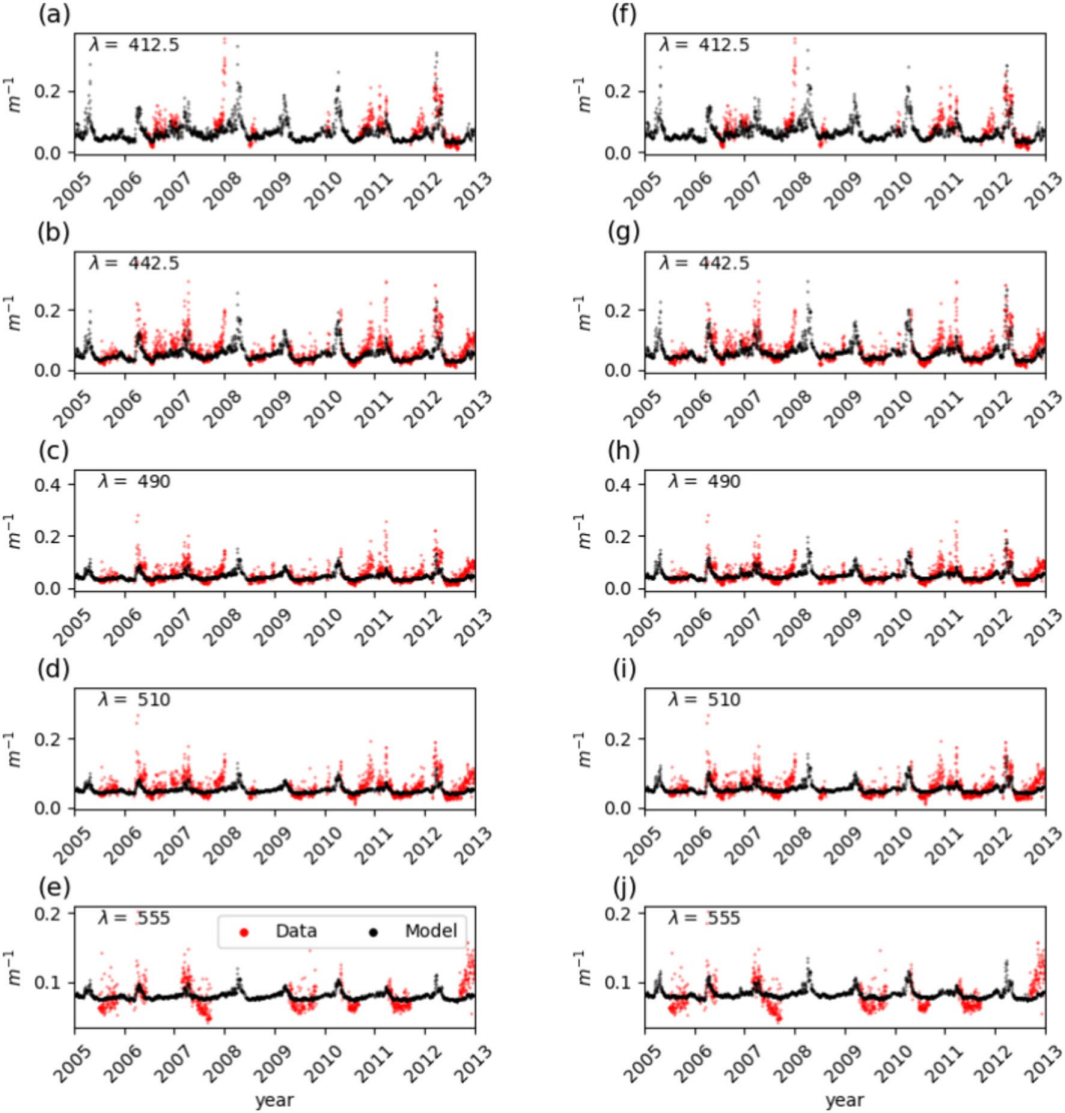
Inversion results of $K_d(\lambda )$ for the years 2005 to 2012 at the BOUSSOLE site. (**a–e**) For the REF configuration and (**f–j**) for the EXP-1-Chl-a configuration; time series of the modelled (black) and in-situ measured (red) spectral diffuse attenuation coefficient at 412.5, 442.5, 490, 510 and 555 nm at daily frequency.

The surface spectral diffuse light attenuation coefficients ($K_d(\lambda )$) also show seasonal variability with maxima in late winter/early spring and low values in summer. In summer, the model tends to overestimate $K_d(412.5)$, Fig. 2a, f, and a similar overestimation of the model is also found for the other wavelengths, Fig. 2. Simulated $K_d(\lambda )$ show higher negative BIAS at the shorter wavelengths, the difference between REF and EXP-1-Chl-a is less pronounced in terms of statistical indicators than the results for Chl-a, except for the BIAS at 442, 490 nm and 510 nm, where EXP-1-Chl-a shows a much lower value.

**Fig. 3:**
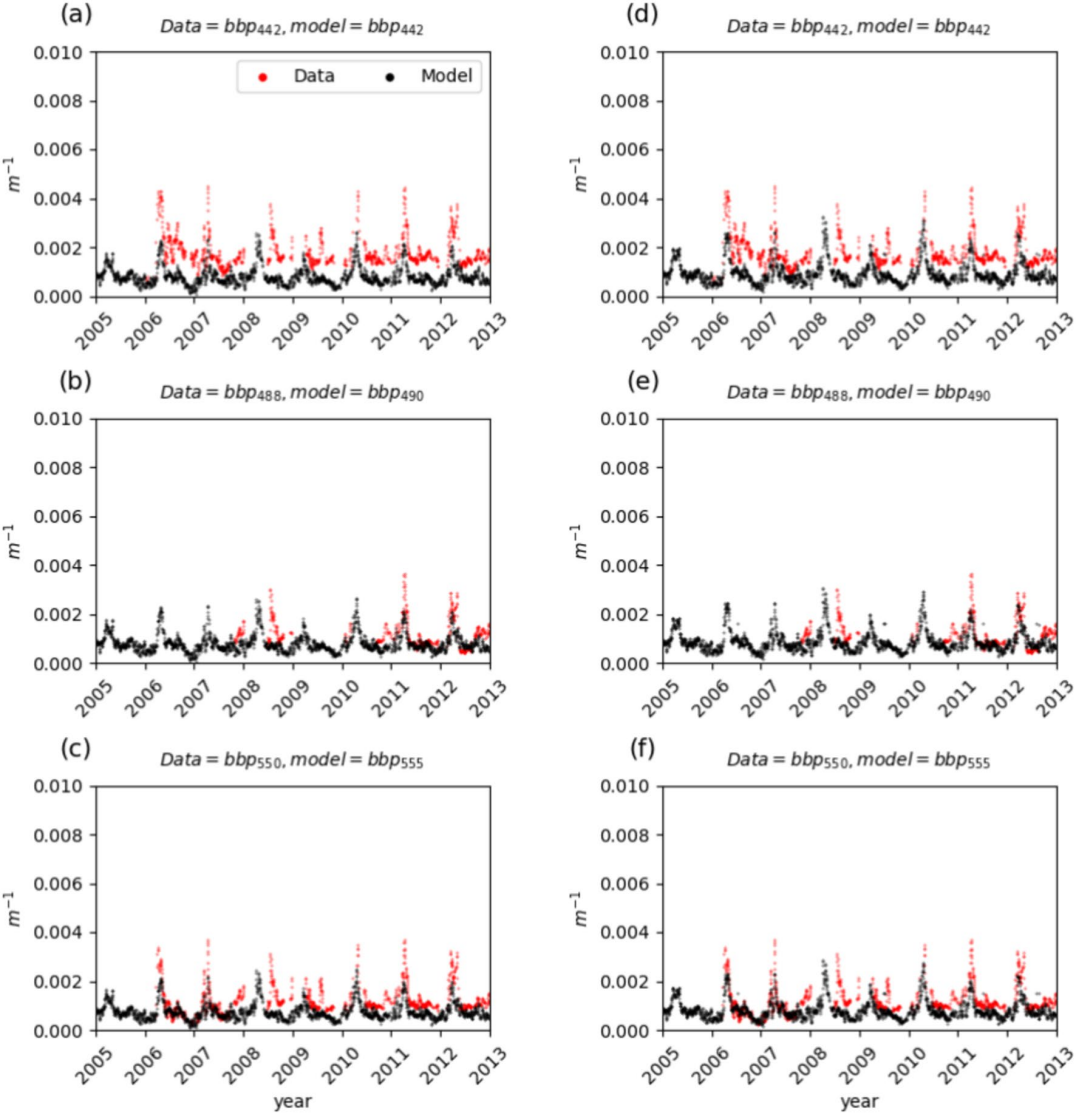
Inversion results of $b_{bp}(\lambda )$ for the years 2005 to 2012 at the BOUSSOLE site. Left panels (**a–c**) for the REF configuration and right panels (**d–f**) for the Exp-1-Chla configuration; time series of modelled (black) and in-situ measured (red) $b_{bp}(442)$, $b_{bp}(490)$ and $b_{bp}(555)$ at daily frequency.

The modeled particle backscattering coefficients are compared with in-situ data in Fig. 3. Particle backscattering, Fig. 3, accounts for both phytoplankton and NAP contributions and shows biologically driven variability with higher backscattering during the spring phytoplankton bloom. In summer, the model tends to underestimate $b_{bp}(442)$, it mathches $b_{bp}(488)$ better and slightly underestimate $b_{bp}(550)$. The BIAS for the particulate backscatter is always negative, Table 1, the correlation is generally lower with respect to Chl-a and $K_d$, and overall the statistical indicators between REF and EXP-1-Chl-a are similar.

### Optimal configurations

The results of the sensitivity experiments show that, depending on the indicator under consideration (Chl-a, $K_{d}$, $b_{bp}$), different parameter configurations provide the best fit. In EXP-1, which analyzes the optical properties of phytoplankton (see Methods section for details), the best parameter configuration has the $a^*_{\rm{PH}}$ used for REF, it has negative slope of $b^*_{\rm{PH}}$ from 412 to 555 *nm* ($b^*_{\rm{PH}}(\lambda =412.5)$/$b^*_{\rm{PH}}(\lambda =555)$=1.57) and lower ratio of backscattering to scattering ($b^*_{b\textrm{PH}}/b^*_{\rm{PH}}=10^{-4}$). The best configurations for $b_{bp}$ at the different wavelengths have the same parameters characteristics: higher absorption than the reference absorption (increase by a factor of 1.9-1.95), negative spectral slope of the scattering ($b^*_{\rm{PH}}(\lambda =412.5)$/$b^*_{\rm{PH}}(\lambda =555)$=22) and low backscattering ($b^*_{b\textrm{PH}}/b^*_{\rm{PH}}=10^{-4}$). In the case of $K_d(\lambda )$, the best set has lower $a^*_{\rm{PH}}$, $b^*_{\rm{PH}}$, $b^*_{b\textrm{PH}}$.

**Fig. 4:**
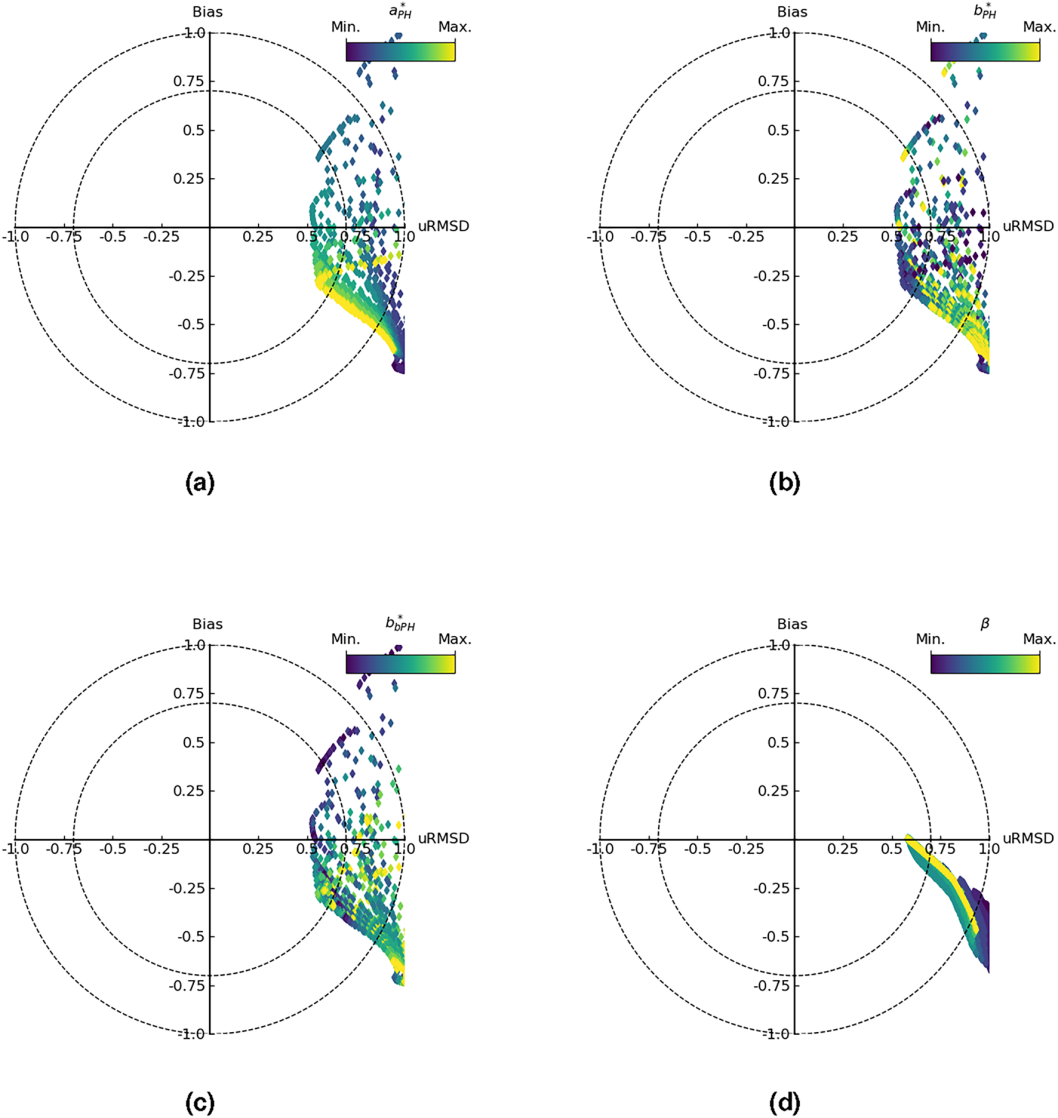
Target diagrams^15^ comparing the results of the model inversion and the in-situ data from Chla-a for the years 2005 to 2012. (**a–c**) The skill metrics for Chl-a from EXP-1, with a color scale referring to the parameter value perturbations $a^*_{\rm{PH}}(\lambda )$, $b^*_{\rm{PH}}(\lambda )$ and $b^*_{b\textrm{PH}}(\lambda )$. (**d**) The skill metrics for Chl-a from EXP-2, with a color scale related to the perturbation of the parameter $\beta$. Details of the parameter perturbations are given in Table 3.

Given the focus of the study, which relates to biogeochemical modeling, the most interesting configuration is the one with the best Chl-a skill metrics. The corresponding statistical metrics, Fig. 4a–c show the dependence of skill on the perturbation of the three parameters $a^*_{\rm{PH}}$, $b^*_{\rm{PH}}$ and $b^*_{b\textrm{PH}}$, respectively. The higher skill is strongly controlled by $a^*_{\rm{PH}}$, Fig. 4a, and $b^*_{b\textrm{PH}}$ given the “stability” of the color near the minimum error with respect to the data (corresponding to the center of the target), and for the same reasons $b^*_{\rm{PH}}$ seems less relevant.

The second group of experiments, EXP-2, appears to be less efficient in terms of error minimization, Fig. 4d. In EXP-2, four parameters are considered and the best configuration for Chl-a is achieved with $\sigma =24~\upmu$ mol Q m$^{-2}$ s$^{-1}$, $\beta =600~\upmu$ mol Q m$^{-2}$ s$^{-1}$, $\theta _0=0.045$ mg Chl mg C$^{-1}$ and $\theta _{min}=0.01$ mg Chl mg C$^{-1}$.

$b_{bp}$ is always better captured with $\theta _0=0.045$ mg Chl mg C$^{-1}$ and $\theta _{min}=0.002$ mg Chl mg C$^{-1}$, but in this case, the chlorophyll patterns are very unrealistic with very low values throughout the year (see the Supplementary Information). Optimal solutions for $K_{d}(\lambda =412.5)$ and $K_{d}(\lambda =442.5)$ show unrealistic Chl-a evolution with very low values throughout the year, while better parameter configurations for $K_{d}(\lambda =412.5,510,555)$ are similar to those with higher skill for Chl-a.

To summarize, EXP-1 with improved parameters for Chl-a (EXP-1-Chl-a) is a good alternative to the REF configuration.

Additional analysis were related to the role of photoprotective pigments. In fact, in principle, the presence of photoprotective pigments also affects the bio-optical properties of the phytoplankton cell, absorption is increased by the presence of additional pigments, such as photoprotective carotenoids (PPC), that protect the photosynthetic machinery against excessive light. We calculated the monthly climatology of the ratio between Chl-a and PPC for the BOUSSOLE site. The analysis of the climatology of available data for the BOUSSOLE site indicates that the most abundant PPC at the site are Zeaxanthin, Violaxanthin, and Diadinoxanthin, see Supplementary Information. The modelling of PPC is less developed than for $\theta _{\rm{CHL}}$ and in this study we considered a monthly variability and normalized the contribution of pigments to absorption with a factor varying from 0 to 1 as its absorption contribution was found to be comparable with respect to the one of Chl-a in high light regimes (i.e. during summer)^16^, but considering only two species grown in culture. Thus, more expanded dataset should be examined. As it was not possible to identify a clear parameterization for such pigments dynamics, we decided to focus only on photoacclimation.

## Discussion

In this study, we investigated and assesed the feasibility of inverting the three-stream irradiance model to derive relevant biogeochemical properties such as Chl-a, NAP, and CDOM absorption coefficient. Semi-analytical methods have been successfully developed in the past^2,17^ and are widely used in remote sensing. This work relies on the formalism and concepts introduced in advanced bio-optical biogeochemical models^6^ and in particular^4^, with the novelty that we consider here the problem of the inversion of the three-stream model. A similar inversion procedure was developed as part of the 2SeaColor two-stream model^18^, which has proven to be extremely effective, especially for turbid waters, and is focused on the derivation of *Kd*. The application proposed here is to be used in the context of biogeochemical modelling, where a forward model is used to calculate light penetration along the water column and an analogous coherent inversion model can be used as an observation operator to assimilate remote sensing reflectance. In this context the inversion model will be applied to open ocean, such as BOUSSOLE site, but simultaneously to more optically complex coastal waters, as for example the northern Adriatic Sea. The study has raised a number of interesting questions.

The use of $R_{\rm{rs}}$ as input data for the inversion approach and, as an extension, for data assimilation requires, like all ocean color applications, a special quality control procedure. The procedure proposed here appears to be well suited to reduce noise in the model output while preserving the temporal and spectral variability of the observational data. It is clear that further work is needed, such as testing the assimilation with a simplified 1D biogeochemical model for the BOUSSOLE site^19^.The application with 3D operational systems, as used in the Copernicus Marine Service (https://marine.copernicus.eu/), may require the further development of quality control procedures to handle the $R_{\rm{rs}}$ data for the assimilation procedure.

From a scientific point of view, the use of an analytical approach in conjunction with sensitivity analysis experiments is useful to understand which are the most critical processes controlling the information flow from measured data, i.e. $R_{\rm{rs}}$, to biogeochemical properties. This approach could be combined with modern machine learning methods such as Physically Informed Neural Networks. The IOPs analysed in EXP-1, such as phytoplankton absorption and particulate backscattering coefficients, appear to be important elements influencing model skill. Physiological processes such as phytoplankton photoacclimation, which affect absorption and backscattering, are also a key element to consider. The analysis of the modelled spectral shape of particle backscattering shows that within the modelling hypotheses, it is difficult to reproduce the decrease of $b_{bp}$ observed at BOUSSOLE, with higher values at 442 nm, which decreases sharply at 490*nm*. We have analysed different NAP constituents but all yield flatter spectral shapes for $b_{bp}$, see Supplementary Information. A possible explanation could be that the scattering of the phytoplankton has a marked clearly negative slope. Indeed, the tests in EXP-1 with the best ability to reconstruct the $b_{bp}$ spectral shape has a higher scattering slope ($b^*_{\rm{PH}}(\lambda =412.5)$/$b^*_{\rm{PH}}(\lambda =555)$=22), but more data on these optical traits of the phytoplankton are needed.

The presented approach can in principle be extended to any number of constituents, e.g., one could include different phytoplankton functional types with type-specific optical properties and Chl-a to carbon ratios. The problem, in this case, could be that the solution space is non-singular and therefore additional constraints should be added to the minimization procedure to obtain a single stable solution.

The approach can be extended to any number of wavelengths, since the functional J defined in Eq. (1), which represents the cost function of the error between the model and the observations, can in principle be iterated over any number of bins, including for example the hyperspectral data streams from PRISMA or PACE satellite sensors^10^.

The dependence of bio-optical and physiological parameters on different ecological regions should also be taken into account. The sensitivity experiments show the impact of parameter perturbations at the BOUSSOLE site, but it would be important to further evaluate how this approach works in other areas where phytoplankton IOPs could be very different: different pigment compositions could influence absorption, or different size distributions could influence the spectral shape of the backscattering coefficient.

## Methods

### Study area

In the present work, in-situ data were acquired at the BOUSSOLE site in the Ligurian Sea ($7^\circ 54$’E, $43^\circ 22$’N), one of the Northwestern Mediterranean sub-basins, at about 32 nautical miles from the French coast (water depth is 2440 *m*), as shown in the Supplementary Information. Here an autonomous fixed buoy is deployed since 2003^20^, sampling bio-optical parameters every 15 min, and monthly oceanographic cruises are conducted since 2001 for discrete bio-optical sampling. The BOUSSOLE site is located in the central area of the cyclonic circulation characterizing the Ligurian Sea where the prevailing ocean currents are weak ($<20$ cm s$^{-1}$). Generally, this site shows a marked seasonality of the physical forcing, switching from deep ($\sim {400}$ m depth) mixed layers in winter to a prevailing stratification in summer ($\sim {20}$ m). Oligotrophic conditions prevail during the summer period with Chl-a values less than $0.1~\hbox {mg}~\hbox {m}^{-3}$ at surface (with minima $\sim 0.05$ mg m$^{-3}$) and undetectable nitrate levels. The early spring phytoplankton bloom period usually occurs from February to March-April, producing higher Chl-a concentrations up to 3–5 mg m$^{-3}$ because of the nitrate repleted surface waters^21^. Most of the other periods of the year are characterized by moderate concentrations of Chl-a, ranging from 0.1 to 0.2 mg m$^{-3}$. Considering these oceanographic conditions, four distinct trophic situations have therefore been identified in the area: vertical winter mixing (November–December–January), spring phytoplankton bloom (February–March–April), summer stratification (May–June–July–August), and fall oligotrophic conditions (September–October).

### Chlorophyll-a and inherent optical properties (IOPs)

Light absorption coeffecients were acquired from monthly cruises. CDOM samples were analyzed and quality-controlled. The particulate absorption spectra were decomposed into phytoplankton ($a_ph({\lambda })$) and non-algal particle ($a_{\rm{NAP}}({\lambda })$; i.e., detritus) absorption coefficients using the numerical decomposition technique^22^.

Phytoplankton pigment concentrations was measured by high performance liquid chromatography (HPLC)^23^. The fluorescence emission of Chlorophyll-a was measured by WETLabs ECOFLNTUs fluorometers at 4 and 9 m at the fixed buoy and calibrated with discrete samples from HPLC analyses^24^, these data were daily averaged to be compared with model output.

The in-situ volume scattering function (VSF) at $140^\circ$, $\beta (140)$, was collected with HOBI Labs (Hydro-Optics, Biology, and Instrumentation Laboratories) Hydroscat-4 backscattering meters installed at the lower measurement depth of the buoy (9 m) and equipped with filters at 442, 488, 550 and 620 nm. From these measurements, the particulate backscattering coefficient was calculated^25^.

### Apparent optical properties (AOPs)

Radiometric data were acquired from the fixed buoy with multispectral Satlantic OCR-200 series at 7 wavelenghts. Downward plane irradiance, $E_d(z,{\lambda })$, was measured at the surface (4.5 m above water), 4 and 9 m depth. Nadir upward radiance $L_u(z,{\lambda })$ was measured at 4 and 9 m depth. Measurements with $E_d(z,{\lambda })$ values lower than $0.005~{\upmu }$W cm$^{-2}$ nm$^{-1}$ were excluded from further analyses. The diffuse attenuation coefficient $K_d(\lambda)$ were calculated from radiometric measurements as in^21^. The remote sensing reflectance, $R_{\rm{rs}}$, was obtained form the Copernicus Marine Data Store (https://scihub.copernicus.eu, multisensor L3 product), and it is used as model input.

To ensure consistency amongst the acquired AOPs, $K_d$ and $R_{\rm{rs}}$, and therefore, facilitate their evaluation, a preliminary phase was performed.

- Quality check (QC) of $K_d$ data: Only $K_d$ data recorded in the 10:00 to 14:00 GMT time window were kept as representative of the measurements for each day. Data acquired with an absolute tilt higher than $10^{\circ }$ were filtered out from the analysis. Measurements collected at a depth of more than 2 *m* below the nominal depth were removed. In fact, observations collected with the above water sensor too close to the sea surface were discarded. Moreover, potential outliers were detected and removed using the 3-$\sigma$ rule over the time average. After visually inspecting all spectra, geophysically unrealistic data or spectra with negative values (e.g., those monotonically increasing magnitudes for the attenuation coefficient) were removed. In addition, the amplitude variability in $K_d$ measurements were smoothed using the Savitzky–Golay (SG) filtering technique, while adopting a polynomial of a third order and a smoothing window size of 10 days. The smoothing action of the SG filter produced the lowest noise while keeping the sharpest step response.
- Quality Control (QC) of satellite $R_{\rm{rs}}$: The $R_{\rm{rs}}$ are the product of a multi-sensor merging of SeaWiFS, MODIS, MERIS, VIIRS-SNPP & JPSS1, and OLCI ocean color sensor mounted on sentinel-3A and sentinel-3B satellites^9^, with a $\sim 1$-day revisit period and at a spatial resolution of $1 \times 1\,\hbox {km}$ (https://scihub.copernicus.eu/). These data were acquired from January 2005 to December 2021 and we focused on the period from 2005 to 2012. The acquired L3 daily multi-sensor $R_{\rm{rs}}$ went through certain QC steps during data treatment. These processes included cloud filtering to remove clouds/shadows from the analysis. This step helps to remove outliers affected by clouds with measurements that do not represent the nature of the target and can lead to misleading results. Potential outliers were removed using the 3-$\sigma$ rule over the quality indicator of $R_{\rm{rs}}$ at each wavelength. At the end, a smoothing process is applied to $R_{\rm{rs}}$ using the method of Savitzky-Golay filtering. This process performs noise reduction while preserving the original spectral features of the spectrum such as absorption band heights and widths. For $R_{\rm{rs}}$ data at each studied wavelength, we least-squares fit a polynomial of a third order and a smoothing window length of 10 days. Before proceeding with this step, a parameter optimization selection phase was carried out to select the optimal polynomnial order and frame size according to the characteristics of the spectrum.

### Prominent features of the satellite $R_{\rm{rs}}$ spectrum

Peaks and troughs in the $R_{\rm{rs}}$ spectra is modulated by the optical properties of oceanic water and at a lesser extent by phytoplankton absorption. Average $R_{\rm{rs}}$ spectra for 2012 shows relatively higher remote sensing reflectance values at the blue end region (412–490 nm), followed by an abrupt downward slope starting at around 500 nm due to water particles absorption, as shown in the Supplementary Information. These values are explained by the theoretical blue absorption peak of Chlorophyll-a at around 443 nm followed by a mild smooth increase to a prominent peak at the the end blue region mainly at 490 nm. This reflectance peak stands for the dominant colour of the water, that is mostly bluish over the entire zone^1^.

### Three-stream light inversion model

**Fig. 5:**
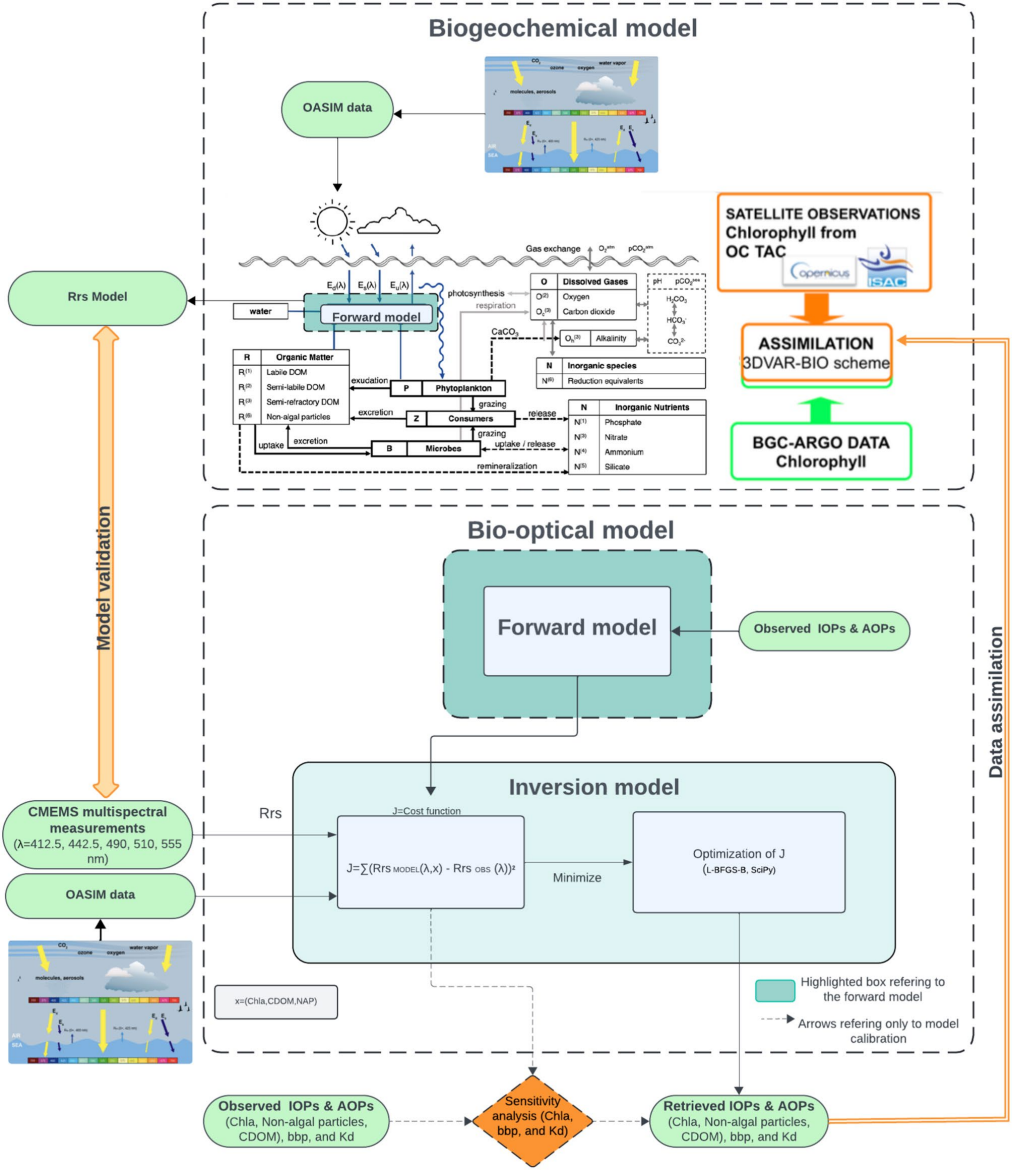
Schematic flowchart of the operational system in which the inversion model will be embedded. The forward model used for the inversion is shared with the operational model currently in operation within the Marine Copernicus Service. The line regarding data assimilation points to future applications where it will be possible to assimilate the biogeochemical variables Chl-a, CDOM, NAP derived with the inversion model alongside the variables that are currently assimilated. A sensitivity procedure can be used to analyze the dependence of model skill on parameter perturbations or to optimize model results for specific regions.

The three-stream method to simulate light propagation in the forward implementation is currently used operationally in the MedBFM system within the Marine Copernicus Service^26^. In this framework, the optical properties such as chlorophyll, CDOM and NAP are used to calculate the remote sensing reflectance. In the present work, we have considered an equivalent model, but used for the opposite task, namely using reflectance data to derive optical properties, Fig. 5. The inversion procedure is based on the minimization of a cost function *J* in the form of a mean square difference depending on the considered optical component, *x*:

1 $$\begin{aligned} J(x) = \sum _{\lambda } \Big ( R_{\rm{rs}}^{\textrm{MODEL}}(\lambda ,x) - R_{\rm{rs}}^{\textrm{OBS}} (\lambda )\Big )^2 \end{aligned}$$

The $x=(\mathrm {Chl-a},\textrm{CDOM},\textrm{NAP})$ vector entries are the concentrations of the biogeochemical parameters determining the modelled reflectances $R_{\rm{rs}}^{\textrm{MODEL}}(\lambda ,x)$. $R_{\rm{rs}}^{\textrm{OBS}}(\lambda )$ are measured remote sensing reflectances. The sum in Eq. (1) spans over the wavelengths of interest measured by the sensors.

The forward light propagation model^4,6^ resolves light propagation according to three streams: a downward direct, sun collimated, component $E_{\rm{dir}}$, a diffuse downward component $E_{\rm{dif}}$ and a diffuse upward component $E_u$.

2 $$\begin{aligned} \frac{dE_{\rm{dir}}(\lambda ,z)}{dz}= & -\frac{a(\lambda )+b(\lambda )}{\cos \theta _d} E_{\rm{dir}}(\lambda ,z) \end{aligned}$$

3 $$\begin{aligned} \frac{dE_{\rm{dif}}(\lambda ,z)}{dz}= & -\frac{a(\lambda )+r_{\rm{dif}}b_{b}(\lambda )}{\overline{v}_{\rm{dif}}}E_{\rm{dif}}(\lambda ,z)+\frac{r_u b_{b}(\lambda )}{\overline{v}_u}E_u(\lambda ,z)+\frac{b(\lambda )-r_{\rm{dir}} b_{b}(\lambda )}{\cos \theta _d}E_{\rm{dir}}(\lambda ,z) \end{aligned}$$

4 $$\begin{aligned} -\frac{dE_u(\lambda ,z)}{dz}= & -\frac{a(\lambda )+r_ub_b(\lambda )}{\overline{v}_u}E_u(\lambda ,z)+\frac{r_{\rm{dif}} b_b(\lambda )}{\overline{v}_s}E_{\rm{dif}}(\lambda ,z)+\frac{r_{\rm{dir}} b_b(\lambda )}{\cos \theta _d}E_{\rm{dir}}(\lambda ,z) \end{aligned}$$

5 $$\begin{aligned} E_{\rm{dir}}(\lambda ,0^-)= & E_{\rm{dir}}^{\textrm{OASIM}}(\lambda ,0^-), ~~~~~ E_{\rm{dif}}(\lambda ,0^-)~~=~~ E_{\rm{dif}}^{\textrm{OASIM}}(\lambda ,0^-), ~~~~~ E_u(\lambda ,\infty ) ~~=~~0 \end{aligned}$$

where $a(\lambda )$, $b(\lambda )$ and $b_{b}(\lambda )$ are the total absorption, scattering and backscattering coefficients, respectively, which are independent of the ambient light field and defined as inherent optical properties (IOPs). $r_{\rm{dir}}$, $r_{\rm{dif}}$ and $r_u$ are the effective scattering coefficients, and $\cos \theta _{\rm{dir}}$, $\overline{v}_{\rm{dif}}$ and $\overline{v}_u$ are the average cosines of the three light fields, which are constant for diffuse irradiance but vary with solar zenith angle for direct irradiance. The boundary conditions at surface, $E_{\rm{dir}}^{\textrm{OASIM}}(\lambda ,0^-)$ and $E_{\rm{dif}}^{\textrm{OASIM}}(\lambda ,0^-)$, are obtained from the OASIM model validated for the BOUSSOLE site^12^. The $R_{\rm{rs},\lambda }^{\textrm{MODEL}}$ is computed as a ratio between the diffuse upward component normalized over the sum of downward components and over the Q factor^4,27^:

6 $$\begin{aligned} R_{rs,\lambda }^{\textrm{MODEL}}=\frac{E_u(\lambda ,0^+)}{Q(\theta _{\rm{dir}})[E_{\rm{dir}}(\lambda ,0^+) + E_{\rm{dif}}(\lambda ,0^+)]} \end{aligned}$$

The transition between the water interface, from just above the sea surface ($0^+$) to just below the sea surface ($0^-$) and the correction for Raman scattering are based on empirical relationships^17,28^.

$a(\lambda )$, $b(\lambda )$, $b_{b}(\lambda )$ are determined summing the product of the each optical constituent concentration and the specific optical coefficients as reported in Table 2, Eqs. 14, 15, and 16 plus the contribution of seawater ($a_{w,\lambda }$, $b_{w,\lambda }$, $b_{b,w,\lambda }$).

7 $$\begin{aligned} a(\lambda )= & a_{w}(\lambda ) + a^*_{\rm{PH}}(\lambda ) \cdot \textrm{Chl}\text { - }\textrm{a} + a^*_{\rm{CDOM}}(\lambda ) \cdot \textrm{CDOM} + a^*_{\rm{NAP}}(\lambda ) \cdot NAP \end{aligned}$$

8 $$\begin{aligned} b(\lambda )= & b_{w}(\lambda ) + b^*_{\rm{PH}}(\lambda ) \cdot \underbrace{\theta _{\rm{CHL}}(\textrm{PAR})^{-1} \cdot \textrm{Chl}\text { - }\textrm{a}}_{C} + b^*_{\rm{NAP}}(\lambda ) \cdot \textrm{NAP} \end{aligned}$$

9 $$\begin{aligned} b_{b}(\lambda )= & b_{bw}(\lambda ) + b^*_{b\textrm{PH}}(\lambda ) \cdot \underbrace{\theta _{\rm{CHL}}(\textrm{PAR})^{-1} \cdot \textrm{Chl}\text { - }\textrm{a}}_{C} + b^*_{b\textrm{NAP}}(\lambda ) \cdot \textrm{NAP} \end{aligned}$$

The relationships between the total coefficients and the biogeochemical properties Chl-a, CDOM, and NAP imply that the light propagation equations are coupled for each wavelength; for example, the change in Chl-a concentration affects all wavelengths. We used the ratio of Chl-a to carbon to express the C concentration of phytoplankton as the product of the reciprocal of $\theta _{\rm{CHL}}(\textrm{PAR})$ by Chl-a to determine the scattering and backscattering of phytoplankton in Eqs. (8) and (9). With this approach the information derived from the inversion of $R_{\rm{rs}}$ for each wavelength can be used simultaneously to determine the vector *x* that is the target of the inversion. An additional constrain makes Chl-a to carbon ratio ($\theta _{CHL}$) dependent on surface irradiance^29^, through a sigmoidal curve:

10 $$\begin{aligned} \theta _{\rm{CHL}}(\textrm{PAR})= & \theta ^{0}_{\rm{CHL}}\frac{e^{-(\textrm{PAR}-\beta )/\sigma }}{1+e^{-(\textrm{PAR}-\beta )/\sigma }} +\theta ^{\textrm{min}}_{\rm{CHL}} \end{aligned}$$

with PAR expressed as $\upmu$ mol Q m$^{-2}$ s$^{-1}$, $\theta ^{0}_{\rm{CHL}}=0.03$ mg Chl mg C$^{-1}$, $\theta ^{\textrm{min}}_{\rm{CHL}}=0.005$ mg Chl mg C$^{-1}$, $\sigma =20 ~ \upmu$ mol Q m$^{-2}$ s$^{-1}$ $\beta = 500 ~ \upmu$ mol Q m$^{-2}$ s$^{-1}$. PAR is computed from OASIM model output integrated from 400 to 700 nm^30^. The choice of a sigmoid function is justified by the assumption that two saturation regimes at low and high light regimes are considered^31^.

To build the inversion tool we assume that the water column is infinitely deep and homogenous^32^. In preliminary tests, we found that, assuming no a-priori correlation between biogeochemical properties at different layers, the error minimization procedure was correcting only the shallower layer contiguous to the boundary condition, therefore we considered only homogeneous concentration of biogeochemical properties along the vartical. In the particular case of the one-layer model the analytical solution of the system of Eqs. (2)–(4) can be derived:

11 $$\begin{aligned} E_{\textrm{dir}}(z)&= E_{\textrm{dir}}(0)\exp \int _0^z (-c_{\rm{dir}}) d\zeta , \end{aligned}$$

12 $$\begin{aligned} E_{\textrm{dif}}(z)&= \big (E_{\rm{dif}}(0) - x E_{\rm{dir}}(0)\big )e^{-k^{+} z} + xE_{\textrm{dir}}(z), \end{aligned}$$

13 $$\begin{aligned} E_u(z)&= \big (E_{\rm{dif}}(0) - x E_{\rm{dir}}(0)\big )r^+e^{-k^{+} z} + yE_{\textrm{dir}}(z). \end{aligned}$$

Here $k^+$, *x*, *y*, $c_{\rm{dir}}$ are functions of the coefficients of the system (2)–(4), as given in the Supplementary Information.

The minimization of the functional *J* is operated using the “limited memory algorithm for bound constrained optimization” L-BFGS-B^33,34^ embedded and freely available within the *Python SciPy package*^35^.

### Optical constituents

The spectral absorption coefficients of seawater $(a_w (\lambda ), \textrm{m}^{-1})$ was taken from literature data^36^. The scattering coefficients of seawater ($b_w(\lambda ), \textrm{m}^{-1}$) was taken from Smith and Baker (1981) and linearly interpolated to the model wavelengths (412.5, 442.5, 490, 510, 555, 560, 665, 670 and 681.25 nm). The backscattering to total scattering ratio of seawater was set to 0.5 ($\tilde{b}_bw$, Morel (1974)). For each non-water constituent, their IOPs were computed as the product of the constituent mass and their respective mass-specific absorption or scattering coefficients. To prescribe the optical properties of phytoplankton we used Chl-a-specific absorption spectra of phytoplankton cultures from several taxa ($a^*_{\rm{PH}}(\lambda )$, m$^2$ mg Chl$^{-1}$) , digitized from literature and provided as Supplementary Information in a specific study focused on the Mediterranean Sea^37^. The collection of $a_{\rm{PH}} (\lambda )$ are originally reported in 6 *nm* intervals from 300 to 800 nm and averaged for four phytoplankton functional types (PFTs) that represented picophytoplankton, nanophytoplankton, diatoms and dinoflagellates. In this work, the four spectra were further averaged and linearly interpolated to the model wavelengths. The carbon specific scattering and backscattering coefficients of phytoplankton ($b^*_{\rm{PH}}(\lambda )~\textrm{and}~ b^*_{\rm{bPH}}(\lambda )$, m$^2$ mg C$^{-1}$) were parameterized as in^4^. Colored dissolved organic matter (CDOM) is an important contributor to total absorbtion but a negligible contribution to scattering. The mass-specific absorption coefficients of CDOM ($a^*_{\rm{CDOM}}(\lambda )$, m$^2$ mg  C$^{-1}$) were considered to decrease exponentially with increasing wavelength as:

14 $$\begin{aligned} a^*_{\rm{CDOM}}(\lambda )=a^*_{\rm{CDOM}} (450) e^{-S_{\rm{CDOM}}(\lambda -450)} \end{aligned}$$

where $a^*_{\rm{CDOM}}(450)$ is the mass-specific absorption coefficient at 450 nm and is set to 0.015 m$^2$ mg C$^{-1}$  ^37^, and $S_{\rm{CDOM}}$ is the spectral slope between 350 and 500 nm that was set to 0.017 nm$^{-1}$  ^37^. Non-algal particles (NAP) absorb and scatter light. The mass-specific absorption coefficients of NAP ($a^*_{NAP}(\lambda )$, m$^2$ mgC$^{-1}$) were considered to decrease exponentially with increasing wavelength^38^, as:

15 $$\begin{aligned} a^*_{\rm{NAP}}(\lambda )=a^*_{\rm{NAP}}(440)e^{-S_{\rm{NAP}} (\lambda -440)} \end{aligned}$$

where $a^*_{NAP}(440)$ is the mass-specific absorption coefficient at 440 nm set to 0.0013 m$^2$ mg C$^{-1}$  ^37^, and $S_{NAP}$ is the spectral slope, set to 0.013 nm$^{-1}$  ^37^. The NAP mass-specific scattering coefficients ($b^*_{NAP} (\lambda )$ m$^2$ mmol C$^{-1}$) were computed as an exponential function of wavelength^38^, as:

16 $$\begin{aligned} b^*_{\rm{NAP}}(\lambda )=b^*_{\rm{NAP}} (550)\left( \frac{550}{\lambda }\right) ^{f_{\rm{NAP}}} \end{aligned}$$

where $b^*_{\rm{NAP}}(550)$ is the mass-specific scattering at 550 *nm* of 0.02875 m$^2$ mg C$^{-1}$ and $f_{\rm{NAP}}$ an exponent of 0.5 nm$^{-1}$  ^37^. The backscattering to total scattering ratio for NAP was set to 0.005, i.e. a scattering efficiency typical of small organic detritus^38^.

**Table 2:** Bio-optical parameters used for water and phytoplankton absorption, scattering and backscattering.

$\lambda$ (nm)	$a_{w}(\lambda )$ (m$^{-1}$)	$b_{w}(\lambda )$ (m$^{-1}$)	$b_{bw}(\lambda )$ (m$^{-1}$)	$a^*_{\rm{PH}}(\lambda )$ (m$^{2}$ mg Chl$^{-1}$)	$b^*_{\rm{PH}}(\lambda )$ (m$^{2}$ mg C$^{-1}$)	$b^*_{b\textrm{PH}}(\lambda )$ (m$^{2}$ mg C$^{-1}$)
412.5	0.0048	0.00535	0.002674	0.034	0.02102	5.38E−05
442.5	0.00742	0.00437	0.002184	0.040	0.02022	5.18E−05
490	0.01758	0.00284	0.001421	0.028	0.02054	5.26E−05
510	0.02918	0.00247	0.001234	0.018	0.02050	5.25E−05
555	0.06098	0.00167	0.000836	0.009	0.01907	4.88E−05

The wavelengths considered are the ones used for the inversion procedures and match with the $R_{\rm{rs}}$ wavelengths measured by satellite sensor data used in the present work.

### Sensitivity experiments

We have considered two classes of ensemble experiments: EXP-1 and EXP-2. The first refers to the optical properties of phytoplankton, the second to the physiological properties of phytoplankton, the perturbed parameters are shown in Table 3. For each experiment, we performed ensemble simulations in which we perturbed the model configuration in a neighbouring region of parameter space around the REF configuration. Statistical indicators such as BIAS and Root Mean Square Error (RMSE) are used to compare the role of each parameter in determining model behaviour and accuracy with respect to the data. The statistics are calculated using independent data in relation to the data used as model input for $R_{\textrm{rs}}(\lambda )$, Chl-a, $K_d(\lambda =412.5)$ and $b_{bp}(\lambda =442)$. To account for the possible variability and uncertainty of the parameters that determine the absorption, scattering and backscattering in relation to the concentration of biogeochemical constituents in EXP-1, we performed an ensemble of 1131 simulations ($11^3$ members). We perturbed three parameters, eleven levels each, related to the optical properties of phytoplankton, but left the optical parameters related to CDOM and NAP unchanged. The spectral shape of the phytoplankton absorption curves shows a maximum in the blue wavelength range from 400 to 555 nm^1^. The curve we adopted as a reference configuration is derived from the aggregation of various spectra related to different phytoplankton groups. In EXP-1, to account for the uncertainty and variability associated to this aggregated variable, we perturbed $a^*_{\textrm{PH}}(\lambda )$ multiplying it by a factor varying from 0.05 to 1.95, thus reducing or amplifying the absorption in the blue bands (around 400 nm) versus the green ones (around 550 nm), with these perturbation $a^*_{\textrm{PH}}(\lambda )$ is in the range 0.0017–0.0663  m $^2$ (mg chl)$^{-1}$, compatible with the one observed in nature, though lower with respect to in-situ measured values obtained in previous studies^39^. Two physiological processes related to phytoplankton cells may be of importance: photoacclimation, expressed as the ratio of Chl-a to carbon ($\theta _{\textrm{CHL}}$), and photoprotection, expressed as the ratio of photoprotective pigments to Chl-a. Both are assumed to be dependent on PAR. Experimental formulations of the ratio of Chl-a to carbon^29,31,40^ are widely used in biogeochemical models where basic physiological processes are considered. In general, these formulas predict higher $\theta _{\textrm{CHL}}$ values at low daily PAR conditions (winter in the present study) and lower values at high daily PAR conditions (summer). We focused on the role of photoacclimation and perturbed in EXP-2 the four parameters that modulate $\theta _{\textrm{CHL}}$ as a function of PAR, for a total of 14641 simulations ($11^4$ members): perturbation of the slope of the curve at the inflection point ($\sigma$), the position of the inflection point ($\beta$), the values of $\theta _{\textrm{CHL}}$ for low and high PAR: $\theta ^{0}_{\textrm{CHL}}$ and $\theta ^{\textrm{min}}_{\textrm{CHL}}$.

**Table 3:** Summary of the perturbed parameters in the experiments carried out in this work.

EXP	Symbol	Range	Units	Reference/description
EXP-1	$a^*_{\textrm{PH}}(\lambda )$	0.05–1.95	–	Eq. (7) Amplification of curve defined in Table 2
EXP-1	$b^*_{\textrm{PH}}(\lambda )$	22–0.5	–	Eq. (8) slope $b^*_{\textrm{PH}}(\lambda =412.5)$/$b^*_{\textrm{PH}}(\lambda =555)$
EXP-1	$b^*_{b\textrm{PH}}(\lambda )$	0.00013–0.005	-	Eq. (9) $b_{b\textrm{PH}}/b_{\textrm{PH}}$
EXP-2	$\sigma$	10–30	$\upmu$ mol Q m$^{-2}$ s$^{-1}$	Eqs. (8), (9), (10)
EXP-2	$\beta$	250–750	$\upmu$ mol Q m$^{-2}$ s$^{-1}$	Eqs. (8), (9), (10)
EXP-2	$\theta _0$	0.015–0.045	mg Chl mg C$^{-1}$	Eqs. (8), (9), (10)
EXP-2	$\theta _{min}$	0.0005–0.01	mg Chl mg C$^{-1}$	Eqs. (8), (9), (10)

For each parameter the range of perturbation was explored with 11 incremental steps of parameter variation. Global perturbations are performed for EXP-1 ($11^3=1331$ members) and for EXP-2 ($11^4=14641$ members).

## Supplementary Information

Supplementary Information.

## Data Availability

Chl a and HPLC data at BOUSSOLE are available from http://www.obs-vlfr.fr/Boussole/html/boussole_data/other_useful_files.php (last access: 1 March 2024). IOP data at BOUSSOLE are available upon request to IMEV. Satellite data are available from the EU Copernicus Marine Service cmems_obs-oc_med_bgc-reflectance_my_l3-multi-1km_P1D at 10.48670/moi-00299 (E.U. Copernicus Marine Service Information, 2022).

## References

[1] Kirk, J. T. O. *Light and Photosynthesis in Aquatic Ecosystems* 2nd ed edn. (Cambridge University Press, 1994).

[2] Werdell, P. J. *et al.* An overview of approaches and challenges for retrieving marine inherent optical properties from ocean color remote sensing. *Prog. Oceanogr.* **160**, 186–212. 10.1016/j.pocean.2018.01.001 (2018). 30573929 10.1016/j.pocean.2018.01.001 PMC6296493

[3] Chandrasekhar, S. Radiative transfer. In *Dover Books on Physics* (Dover Publications, 1950).

[4] Dutkiewicz, S. *et al.* Capturing optically important constituents and properties in a marine biogeochemical and ecosystem model. *Biogeosciences* **12**, 4447–4481. 10.5194/bg-12-4447-2015 (2015).

[5] Cossarini, G. *et al.* High-resolution reanalysis of the mediterranean sea biogeochemistry (1999–2019). *Front. Mar. Sci.* **8**, 741486. 10.3389/fmars.2021.741486 (2021).

[6] Gregg, W. W. & Rousseaux, C. S. Simulating PACE global ocean radiances. *Front. Mar. Sci.*[SPACE]10.3389/fmars.2017.00060 (2017). 29292403 10.3389/fmars.2017.00060 PMC5747546

[7] Baird, M. E. *et al.* Remote-sensing reflectance and true colour produced by a coupled hydrodynamic, optical, sediment, biogeochemical model of the great barrier reef, Australia: Comparison with satellite data. *Environ. Model. Softw.* **78**, 79–96. 10.1016/j.envsoft.2015.11.025 (2016).

[8] Jones, E. M. *et al.* Use of remote-sensing reflectance to constrain a data assimilating marine biogeochemical model of the great barrier reef. *Biogeosciences* **13**, 6441–6469. 10.5194/bg-13-6441-2016 (2016).

[9] Donlon, C. *et al.* The global monitoring for environment and security (GMES) Sentinel-3 mission. *Remote Sens. Environ.* **120**, 37–57. 10.1016/j.rse.2011.07.024 (2012).

[10] Loizzo, R. *et al.* Prisma mission status and perspective. In *IGARSS 2019—2019 IEEE International Geoscience and Remote Sensing Symposium*. 4503–4506 10.1109/IGARSS.2019.8899272 (IEEE, 2019).

[11] Werdell, P. J. *et al.* The plankton, aerosol, cloud, ocean ecosystem mission: Status, science, advances. *Bull. Am. Meteorol. Soc.* **100**, 1775–1794. 10.1175/BAMS-D-18-0056.1 (2019).

[12] Lazzari, P. *et al.* CDOM spatiotemporal variability in the Mediterranean sea: A modelling study. *J. Mar. Sci. Eng.* **9**, 176. 10.3390/jmse9020176 (2021).

[13] Dowd, M., Jones, E. & Parslow, J. A statistical overview and perspectives on data assimilation for marine biogeochemical models: Overview of marine biogeochemical data assimilation. *Environmetrics* **25**, 203–213. 10.1002/env.2264 (2014).

[14] Antoine, D. *et al.* Boussole: A joint CNRS-INSU, ESA, CNES, and NASA ocean color calibration and validation activity. In *Technical Report*, National Aeronautics and Space Administration (2006).

[15] Jolliff, J. K. *et al.* Summary diagrams for coupled hydrodynamic-ecosystem model skill assessment. *J. Mar. Syst.* **76**, 64–82. 10.1016/j.jmarsys.2008.05.014 (2009).

[16] Moore, L. R., Goericke, R. & Chisholm, S. W. Comparative physiology of synechococcus and prochlorococcus: Influence of light and temperature on growth, pigments, fluorescence and absorptive properties. *Mar. Ecol. Prog. Ser.* **116**, 259–275 (1995).

[17] Lee, Z., Carder, K. L. & Arnone, R. A. Deriving inherent optical properties from water color: A multiband quasi-analytical algorithm for optically deep waters. *Appl. Opt.* **41**, 5755. 10.1364/AO.41.005755 (2002). 12269575 10.1364/ao.41.005755

[18] Salama, M. S. & Verhoef, W. Two-stream remote sensing model for water quality mapping: 2SeaColor. *Remote Sens. Environ.* **157**, 111–122. 10.1016/j.rse.2014.07.022 (2015).

[19] Álvarez, E. *et al.* Chromophoric dissolved organic matter dynamics revealed through the optimization of an optical-biogeochemical model in the NW Mediterranean Sea. Preprint. Biogeochemistry: Bio-Optics. 10.5194/bg-2023-48 (2023).

[20] Antoine, D. *et al.* The “Boussole’’ buoy—A new transparent-to-swell taut mooring dedicated to marine optics: Design, tests, and performance at sea. *J. Atmos. Ocean. Technol.* **25**, 968–989 (2008).

[21] Antoine, D. *et al.* Assessment of uncertainty in the ocean reflectance determined by three satellite ocean color sensors (meris, seawifs and modis-a) at an offshore site in the Mediterranean sea (Boussole project). *J. Geophys. Res. Oceans* **113** (2008).

[22] Bricaud, A. & Stramski, D. Spectral absorption coefficients of living phytoplankton and nonalgal biogenous matter: A comparison between the peru upwelling areaand the sargasso sea. *Limnol. Oceanogr.* **35**, 562–582 (1990).

[23] Ras, J., Claustre, H. & Uitz, J. Spatial variability of phytoplankton pigment distributions in the subtropical south Pacific Ocean: Comparison between in situ and predicted data. *Biogeosciences* **5**, 353–369 (2008).

[24] Ciancia, E. *et al.* Quantifying the variability of phytoplankton blooms in the NW Mediterranean sea with the robust satellite techniques (RST). *Remote Sens.* **13**, 5151 (2021).

[25] Antoine, D. *et al.* Variability in optical particle backscattering in contrasting bio-optical oceanic regimes. *Limnol. Oceanogr.* **56**, 955–973 (2011).

[26] Coppini, G. *et al.* The Mediterranean forecasting system—Part 1: Evolution and performance. *Ocean Sci.* **19**, 1483–1516. 10.5194/os-19-1483-2023 (2023).

[27] Aas, E. & Højerslev, N. K. Analysis of underwater radiance observations: Apparent optical properties and analytic functions describing the angular radiance distribution. *J. Geophys. Res. Oceans* **104**, 8015–8024. 10.1029/1998JC900088 (1999).

[28] Lee, Z. *et al.* Penetration of UV-visible solar radiation in the global oceans: Insights from ocean color remote sensing: Penetration of UV-visible solar light. *J. Geophys. Res. Oceans* **118**, 4241–4255. 10.1002/jgrc.20308 (2013).

[29] Cloern, J. E., Grenz, C. & Vidergar-Lucas, L. An empirical model of the phytoplankton chlorophyll : Carbon ratio-the conversion factor between productivity and growth rate. *Limnology and Oceanography* **40**, 1313–1321. 10.4319/lo.1995.40.7.1313 (1995).

[30] Lazzari, P. *et al.* Assessment of the spectral downward irradiance at the surface of the Mediterranean sea using the radiative ocean-atmosphere spectral irradiance model (OASIM). *Ocean Sci.* **17**, 675–697. 10.5194/os-17-675-2021 (2021).

[31] Geider, R., MacIntyre, H. & Kana, T. Dynamic model of phytoplankton growth and acclimation: Responses of the balanced growth rate and the chlorophyll a:carbon ratio to light, nutrient-limitation and temperature. *Mar. Ecol. Prog. Ser.* **148**, 187–200. 10.3354/meps148187 (1997).

[32] Ronald, J. & Zaneveld, V. Remotely sensed reflectance and its dependence on vertical structure: A theoretical derivation. *Appl. Opt.* **21**, 4146. 10.1364/AO.21.004146 (1982). 20401021 10.1364/AO.21.004146

[33] Byrd, R. H., Lu, P., Nocedal, J. & Zhu, C. A limited memory algorithm for bound constrained optimization. *SIAM J. Sci. Comput.* **16**, 1190–1208. 10.1137/0916069 (1995).

[34] Zhu, C., Byrd, R. H., Lu, P. & Nocedal, J. Algorithm 778: L-BFGS-B: Fortran subroutines for large-scale bound-constrained optimization. *ACM Trans. Math. Softw.* **23**, 550–560. 10.1145/279232.279236 (1997).

[35] Virtanen, P. *et al.* SciPy 1.0: Fundamental algorithms for scientific computing in Python. *Nat. Methods* **17**, 261–272 10.1038/s41592-019-0686-2 (2020). 10.1038/s41592-019-0686-2 PMC7056644 32015543

[36] Pope, R. M. & Fry, E. S. Absorption spectrum (380–700 nm) of pure water II integrating cavity measurements. *Appl. Opt.* **36**, 8710. 10.1364/AO.36.008710 (1997). 18264420 10.1364/ao.36.008710

[37] Álvarez, E., Lazzari, P. & Cossarini, G. Phytoplankton diversity emerging from chromatic adaptation and competition for light. *Prog. Oceanogr.* **204**, 102789. 10.1016/j.pocean.2022.102789 (2022).

[38] Gallegos, C. L., Werdell, P. J. & McClain, C. R. Long-term changes in light scattering in Chesapeake Bay inferred from Secchi depth, light attenuation, and remote sensing measurements. *J. Geophys. Res. Oceans* **116**, 2011JC007160. 10.1029/2011JC007160 (2011).

[39] Bricaud, A., Babin, M., Morel, A. & Claustre, H. Variability in the chlorophyll-specific absorption coefficients of natural phytoplankton: Analysis and parameterization. *J. Geophys. Res. Oceans* **100**, 13321–13332. 10.1029/95JC00463 (1995).

[40] Flynn, K. J. A mechanistic model for describing dynamic multi-nutrient, light, temperature interactions in phytoplankton. *J. Plankton Res.* **23**, 977–997. 10.1093/plankt/23.9.977 (2001).

