# Supplementary material for: Determination of biogeochemical properties in sea waters using the inversion of the three-stream irradiance model
Source: Sci Rep. 2024 Sep 27;14:22347. doi: 10.1038/s41598-024-71457-5 (PMC11437028; doi:10.1038/s41598-024-71457-5)
Supplement: Supplementary file 1 — Supplementary Information. [file 41598_2024_71457_MOESM1_ESM.pdf]

Supplementary Information - Determination of Biogeochemical Properties in Sea Waters Using the Inversion of the Three-stream Irradiance Model.

Paolo Lazzari[1,*], Mirna Gharbi Dit Kacem[1,2], Eva Álvarez[1], Ilya Chernov[3], and Vincenzo Vellucci[4,5]

[1]National Institute of Oceanography and Applied Geophysics - OGS, Trieste, I-34014 , Italy
[2]Dipartimento di Matematica e Geoscienze, Università degli Studi di Trieste, Via Valerio 12, Trieste, I-34127, Italy
[3]Institute of Applied Mathematical Research of the Karelian Research Centre of the Russian Academy of Sciences, 185910, Petrozavodsk Russia
[4]Sorbonne Université, CNRS, Institut de la Mer de Villefranche, IMEV, Villefranche-sur-Mer, F-06230, France
[5]Sorbonne Université, CNRS, OSU Station Marines, STAMAR, Paris, F-75006, France
[*]plazzari@ogs.it

Contents

S1 Study site and Rrs input data at BOUSSOLE S2
S2 Satellite and model $R_{\mathrm{rs}}(\lambda)$ S4
S3 Three-stream light inversion model S6
S4 Miniziation algorithm S8
S5 Analysis for NAP backscattering from avaialble data at BOOUSSOLE S8
S6 Sensitivity analysis results S10
S7 Photoprotective pigments climatology at BOUSSOLE S26
References S27

S1 Study site and Rrs input data at BOUSSOLE

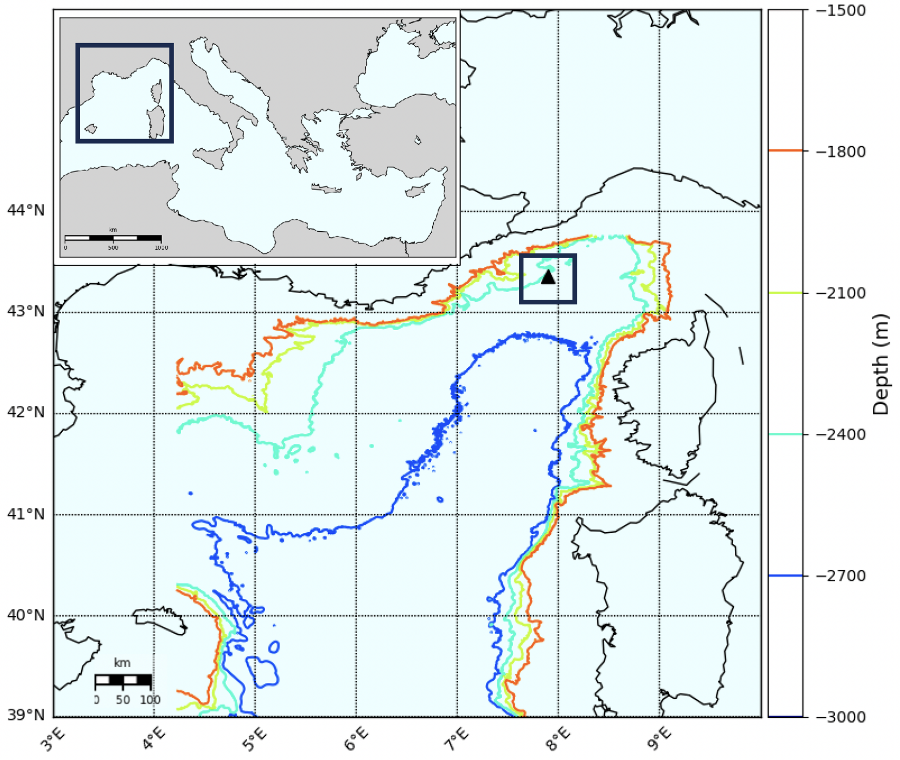

Figure S1. Map of the location of the BOUSSOLE site (Black triangle) in the Ligurian sea Northwestern Mediterranean sub-basin. The graphic was created with Cartopy python package[1].

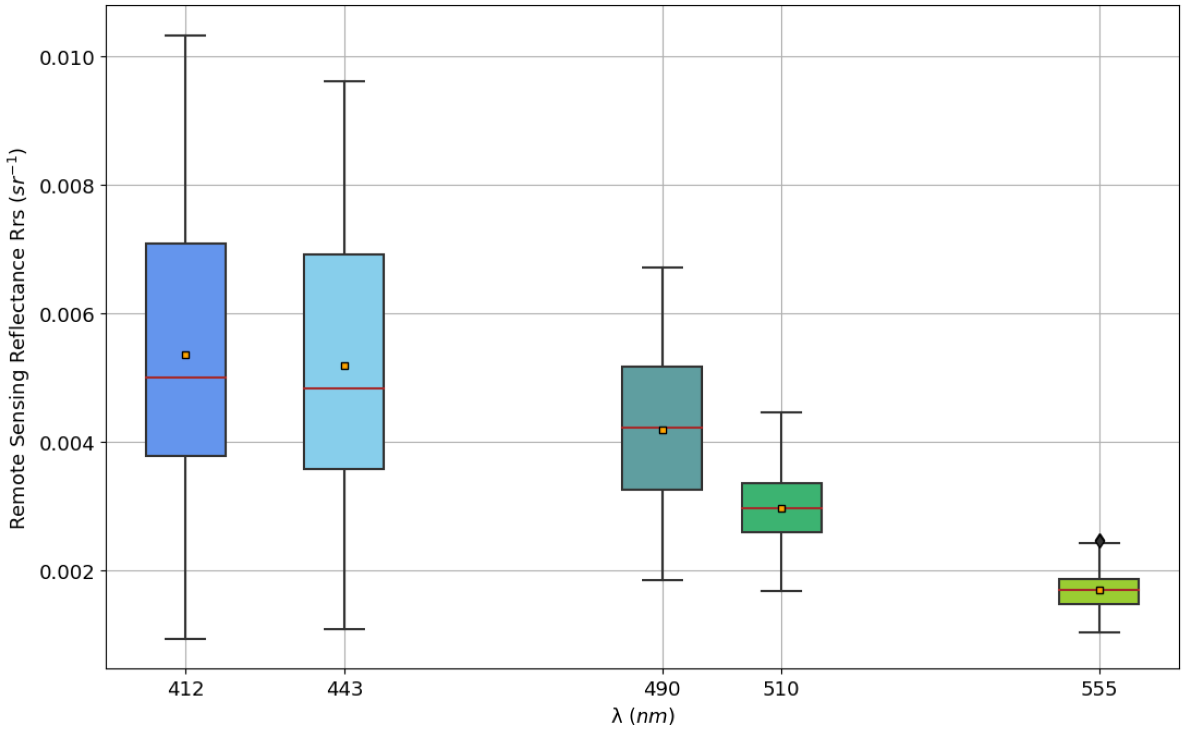

Figure S2. Boxplots of the satellite derived $R_{rs}(\lambda)$ data for the year 2012. Boxes denote the interquartile range at each region of the visible spectrum considered here, colors changes with wavelength. The red lines and the orange rectangles are respectively the median and average in-situ $R_{rs}(\lambda)$ for the year 2012 at the BOUSSOLE site. Black diamonds (♦) denote outliers.

S2 Satellite and model $R_{\mathrm{rs}}(\lambda)$

In this section we show the comparison of Satellite vs model reconstructed $R_{\mathrm{rs}}(\lambda)$. We consider two configurations the REF one, Fig.S3 and the EXP1-Chla providing the best skill on Chl-a, Fig.S4.

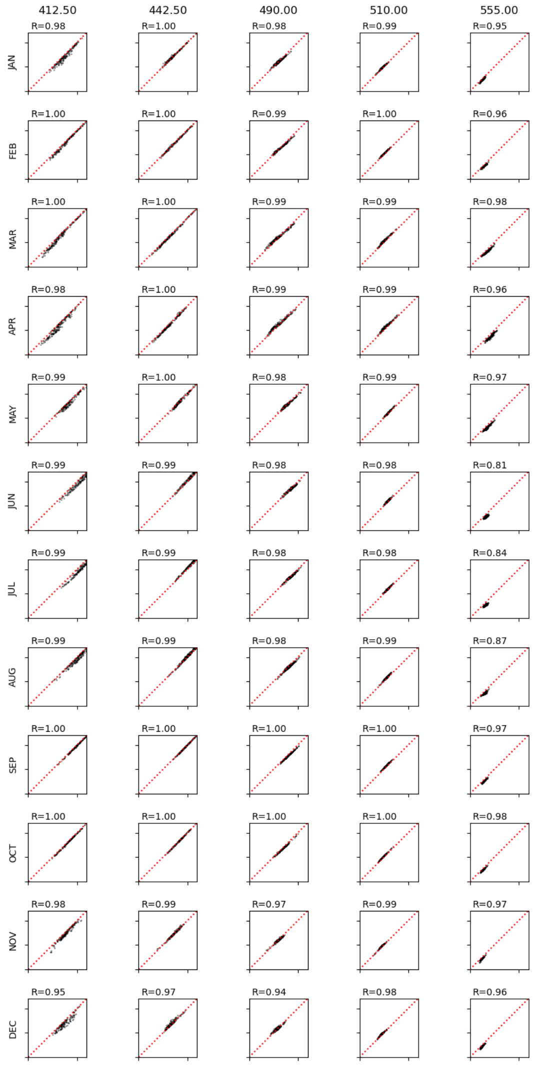

Figure S3. REF model configuration. Comparison of inversion results for $R_{\mathrm{rs}}(\lambda)$, with satellite data on x-axis and modelled data on y-axis, both axis ranges from 0 to 0.012 st^{-1} in all subplots. Data for the period 2005-2012 are aggregated per month in rows and wavelengths in columns.The dotted red line is the 1:1 bisection line.

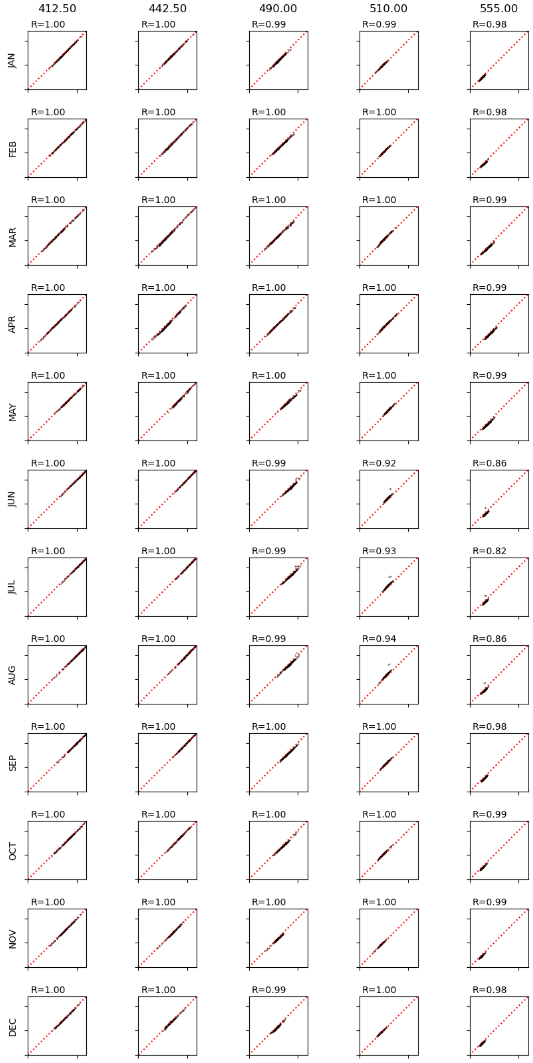

Figure S4. EXP-1-Chla model configuration. Comparison of inversion results for $R_{rs}(\lambda)$, with satellite data on x-axis and modelled data on y-axis, both axis ranges from 0 to 0.012 st^{-1} in all subplots. Data for the period 2005-2012 are aggregated per month in rows and wavelengths in columns.The dotted red line is the 1:1 bisection line.

S3 Three-stream light inversion model

The inversion procedure is based on the minimization of a cost function J in the form of a mean square difference depending on the considered optically active component, x:

$$J(x) = \sum_{\lambda} \left(R_{\text{rs}}^{\text{MODEL}}(\lambda, x) - R_{\text{rs}}^{\text{OBS}}(\lambda) \right)^2 \tag{S.1}$$

The $x = (\text{Chl - a}, \text{CDOM}, \text{NAP})$ vector entries are the concentrations of the biogeochemical parameters determining the modelled reflectances $R_{\text{rs}}^{\text{MODEL}}(\lambda, x)$. $R_{\text{rs}}^{\text{OBS}}(\lambda)$ are measured remote sensing reflectances. The sum in Eq.(S.1) spans over the wavelengths of interest measured by the sensors.

The forward light propagation model[2,3] resolves light propagation according to three streams: a downward direct, sun collimated, component E_{dir}, a diffuse downward component E_{dif} and a diffuse upward component E_u.

$$\frac{dE_{\text{dir}}(\lambda,z)}{dz} = -\frac{a(\lambda)+b(\lambda)}{\cos\theta_d} E_{\text{dir}}(\lambda,z) \tag{S.2}$$

$$\frac{dE_{\text{dif}}(\lambda,z)}{dz} = -\frac{a(\lambda)+r_{\text{dif}} b_b(\lambda)}{\bar{v}_{\text{dif}}} E_{\text{dif}}(\lambda,z) + \frac{r_u b_b(\lambda)}{\bar{v}_u} E_u(\lambda,z) + \frac{b(\lambda) - r_{\text{dir}} b_b(\lambda)}{\cos\theta_d} E_{\text{dir}}(\lambda,z) \tag{S.3}$$

$$-\frac{dE_u(\lambda,z)}{dz} = -\frac{a(\lambda)+r_u b_b(\lambda)}{\bar{v}_u} E_u(\lambda,z) + \frac{r_{\text{dif}} b_b(\lambda)}{\bar{v}_s} E_{\text{dif}}(\lambda,z) + \frac{r_{\text{dir}} b_b(\lambda)}{\cos\theta_d} E_{\text{dir}}(\lambda,z) \tag{S.4}$$

$$E_{\text{dir}}(\lambda,0^-) = E_{\text{dir}}^{\text{OASIM}}(\lambda,0^-), \quad E_{\text{dif}}(\lambda,0^-) = E_{\text{dif}}^{\text{OASIM}}(\lambda,0^-), \quad E_u(\lambda,\infty) = 0 \tag{S.5}$$

where $a(\lambda)$, $b(\lambda)$ and $b_b(\lambda)$ are the total absorption, scattering and backscattering coefficients, respectively, which are independent of the ambient light field and defined as inherent optical properties (IOPs). r_{dir}, r_{dif} and r_u are the effective scattering coefficients, and $\cos\theta_{\text{dir}}$, $\bar{v}_{\text{dif}}$ and $\bar{v}_u$ are the average cosines of the three light fields, which are constant for diffuse irradiance but vary with solar zenith angle for direct irradiance. The boundary conditions at surface, $E_{\text{dir}}^{\text{OASIM}}(\lambda,0^-)$ and $E_{\text{dif}}^{\text{OASIM}}(\lambda,0^-)$, are obtained from the OASIM model validated for the BOUSSOLE site[4]. The $R_{\text{rs},\lambda}^{\text{MODEL}}$ is computed as a ratio between the diffuse upward component normalized over the sum of downward components and over the Q factor[3,5]:

$$R_{rs,\lambda}^{\text{MODEL}} = \frac{E_u(\lambda,0^+)}{Q(\theta_{\text{dir}})[E_{\text{dir}}(\lambda,0^+) + E_{\text{dif}}(\lambda,0^+)]} \tag{S.6}$$

The transition between the water interface, from just above the sea surface (0^+) to just below the sea surface (0^-) and the correction for Raman scattering are based on empirical relationships[6,7].

$a(\lambda)$, $b(\lambda)$, $b_b(\lambda)$ are determined summing the product of the each optically active constituent concentration and the specific optical coefficients as reported in the main text, plus the contribution of seawater ($a_{w,\lambda}$, $b_{w,\lambda}$, $b_{b,w,\lambda}$).

$$a(\lambda) = a_w(\lambda) + a^*_{\text{PH}}(\lambda) \cdot \text{Chl - a} + a^*_{\text{CDOM}}(\lambda) \cdot \text{CDOM} + a^*_{\text{NAP}}(\lambda) \cdot NAP \tag{S.7}$$

$$b(\lambda) = b_w(\lambda) + b^*_{\text{PH}}(\lambda) \cdot \underbrace{\theta_{\text{CHL}}(\text{PAR})^{-1} \cdot \text{Chl - a}}_{C} + b^*_{\text{NAP}}(\lambda) \cdot \text{NAP} \tag{S.8}$$

$$b_b(\lambda) = b_{bw}(\lambda) + b^*_{b\text{PH}}(\lambda) \cdot \underbrace{\theta_{\text{CHL}}(\text{PAR})^{-1} \cdot \text{Chl - a}}_{C} + b^*_{b\text{NAP}}(\lambda) \cdot \text{NAP} \tag{S.9}$$

The relationships between the total coefficients and the biogeochemical properties Chl-a, C, CDOM, and NAP imply that the light propagation equations are coupled for each wavelength; for example, the change in Chl-a concentration affects all wavelengths. This means that the information derived from the inversion of R_{rs} for each wavelength can be used simultaneously to determine the vector x that is the target of the inversion. An additional constrain makes Chl-a to carbon ratio (θ_{CHL}) dependent on surface irradiance[8], through a sigmoidal curve:

$$\theta_{\text{CHL}}(\text{PAR}) = \theta^0_{\text{CHL}} \frac{e^{-(\text{PAR}-\beta)/\sigma}}{1+e^{-(\text{PAR}-\beta)/\sigma}} + \theta^{\text{min}}_{\text{CHL}} \tag{S.10}$$

with PAR expressed as $\mu mol\ Q\ m^{-2}\ s^{-1}$, $\theta^0_{\text{CHL}} = 0.03\ mg\ Chl\ mg\ C^{-1}$, $\theta^{\text{min}}_{\text{CHL}} = 0.005\ mg\ Chl\ mg\ C^{-1}$, $\sigma = 20\ \mu mol\ Q\ m^{-2}\ s^{-1}$ $\beta = 500\ \mu mol\ Q\ m^{-2}\ s^{-1}$. PAR is computed from OASIM model output integrated from 400 to 700 nm[9]. The choice of a sigmoid function is justified by the assumption that two saturation regimes at low and high light regimes are considered[10].

To build the inversion tool we assume that the water column is infinitely deep and homogenous[11]. The inversion approach considering several inhomogeneous layers showed no particular advantage with respect to the one-layer approach used here. In fact, in preliminary tests, we found that, assuming no a-priori correlation between biogeochemical properties at different layers, the error minimization procedure was correcting only the shallower layer contiguous to the boundary condition. In the particular case of the one-layer model the analytical solution of the system of Eq. (S.2)–(S.4) can be derived:

$$E_{\rm dir}(z) = E_{\rm dir}(0)\exp\int_0^z(-c_{\rm dir})d\zeta, \tag{S.11}$$

$$E_{\rm dif}(z) = \big(E_{\rm dif}(0) - xE_{\rm dir}(0)\big)e^{-k^+z} + xE_{\rm dir}(z), \tag{S.12}$$

$$E_u(z) = \big(E_{\rm dif}(0) - xE_{\rm dir}(0)\big)r^+e^{-k^+z} + yE_{\rm dir}(z). \tag{S.13}$$

Here k^+, x, y, $c_{\rm dir}$ are functions of the coefficients of the system (S.2)–(S.4), as given in the following section.

The minimization of the functional J is operated using the "limited memory algorithm for bound constrained optimization L-BFGS-B[12,13] embedded and freely available within the *Python SciPy package*[14].

The analytical solution

The solution presented here integrates the solution[3] taking into account the case of the zero determinant for the inhomogeneous solution and concentrates on the vertically homogeneous water column with infinite depth. The system (S.2)–(S.4) contains one independent linear equation (S.2) and a 2×2 subsystem (S.3)–(S.4). Equation (S.2) can be integrated:

$$E_{\rm dir}(z) = E_{\rm dir}(0)\exp\int_0^z\frac{-(a(\lambda)+b(\lambda))}{\cos\theta_{\rm dir}}d\zeta = E_{\rm dir}(0)\exp\int_0^z(-c_{\rm dir})d\zeta. \tag{S.14}$$

To solve equations (S.3)–(S.4), we write them in the matrix form:

$$\frac{d\mathbf{E}}{dz} = \mathbf{ME}+\mathbf{I}, \quad \mathbf{M} = \begin{bmatrix}-C_{\rm dif} & B_u\\ -B_{\rm dif} & C_u\end{bmatrix}, \ \mathbf{E} = \begin{bmatrix}E_{\rm dif}\\ E_u\end{bmatrix}, \ \mathbf{I} = \begin{bmatrix}F_{\rm dir}\\ -B_{\rm dir}\end{bmatrix}E_{\rm dir}. \tag{S.15}$$

The inhomogeneous solution is searched for in the form

$$\mathbf{E} = \begin{bmatrix}x\\ y\end{bmatrix}E_{\rm dir}, \quad \text{so} \quad -c_{\rm dir}\mathbf{E} = \mathbf{ME}+\mathbf{I}, \quad \begin{bmatrix}-C_{\rm dif}+c_{\rm dir} & B_u\\ -B_{\rm dif} & C_u+c_{\rm dir}\end{bmatrix}\begin{bmatrix}x\\ y\end{bmatrix} = \begin{bmatrix}-F_{\rm dir}\\ B_{\rm dir}\end{bmatrix}. \tag{S.16}$$

The solution of the inhomogeneous equation is obtained inverting the matrix:

$$\begin{bmatrix}x\\ y\end{bmatrix} = \frac{1}{(c_{\rm dir}-C_{\rm dif})(c_{\rm dir}+C_u)+B_{\rm dif}B_u}\begin{bmatrix}C_u+c_{\rm dir} & -B_u\\ B_{\rm dif} & -C_{\rm dif}+c_{\rm dir}\end{bmatrix}\begin{bmatrix}-F_{\rm dir}\\ B_{\rm dir}\end{bmatrix} \tag{S.17}$$

In the singular case $\det\big(\mathbf{ME}+c_{\rm dir}\mathbf{E}\big)=0$, we look for the inhomogeneous solution in the form

$$\mathbf{E} = \begin{bmatrix}x\\ y\end{bmatrix}z\cdot E_{\rm dir}, \quad \text{which yields} \quad \mathbf{E} = \begin{bmatrix}F_{\rm dir}\\ -B_{\rm dir}\end{bmatrix}z\cdot E_{\rm dir}.$$

So, use $zE_{\rm dir}(z)$ instead of $E_{\rm dir}(z)$ in what follows in case the degenerate case (of zero determinant).

To compute the homogeneous solution we need the eigenvalues of $\mathbf{M}$:

$$k^- = D - C_{\rm dif}, \quad -k^+ = C_u - D = -C_{\rm dif} + \frac{B_{\rm dif}B_u}{D}, \quad D = \frac{1}{2}\left(C_{\rm dif}+C_u+\sqrt{(C_{\rm dif}+C_u)^2-4B_{\rm dif}B_u}\right). \tag{S.18}$$

In the homogeneous computational layer the solution is:

$$\begin{bmatrix}E_{\rm dif}(z)\\ E_u(z)\end{bmatrix} = c^+\begin{bmatrix}1\\ r^+\end{bmatrix}e^{-k^+z} + c^-\begin{bmatrix}r^-\\ 1\end{bmatrix}e^{k^-(z-\infty)} + \begin{bmatrix}x\\ y\end{bmatrix}E_{\rm dir}(z), \quad r^+ = \frac{B_{\rm dif}}{D}, \quad r^- = \frac{B_u}{D}. \tag{S.19}$$

which is simplified to

$$\begin{bmatrix}E_{\rm dif}(z)\\ E_u(z)\end{bmatrix} = c^+\begin{bmatrix}1\\ r^+\end{bmatrix}e^{-k^+z} + \begin{bmatrix}x\\ y\end{bmatrix}E_{\rm dir}(z), \tag{S.20}$$

Using the boundary condition, we determine the constant:

$$c^+ = E_{\text{dif}}^{OASIM}(\lambda, 0^-) - xE_{\text{dir}}^{OASIM}(\lambda, 0^-) \quad \text{(in the degenerate case} \quad c^+ = E_{\text{dif}}^{OASIM}(\lambda, 0^-)). \tag{S.21}$$

So, we have the following algorithm. Given

$$c_{\text{dir}} = \frac{a(\lambda)+b(\lambda)}{\cos\theta_d}, \quad C_{\text{dif}} = \frac{a(\lambda)+r_s b_b(\lambda)}{\bar{v}_s}, \quad C_u = \frac{a(\lambda)+r_u b_b(\lambda)}{\bar{v}_u}, \quad B_u = \frac{r_u b_b(\lambda)}{\bar{v}_u}, \quad B_{\text{dif}} = \frac{r_s b_b(\lambda)}{\bar{v}_s},$$
$$F_{\text{dir}} = \frac{b(\lambda)-r_d b_b(\lambda)}{\cos\theta_d}, \quad B_{\text{dir}} = \frac{r_d b_b(\lambda)}{\cos\theta_d},$$

write down the explicit expression for E_{dir} using (S.14). Get x and y using formulae (S.17) and write down the partial solution $\mathbf{E} = [x,y]^T E_{\text{dir}}$. If the matrix M is degenerate, $x = zF_{\text{dir}}$, $y = -zB_{\text{dir}}$. Now, get the eigenvalues k^+, k^- using (S.18). Get the constant c^+ using (S.21) and $r^+ = B_{\text{dif}}/D$ and, finally, write down the solution:

$$E_{\text{dir}}(z) = E_{\text{dir}}(0)\exp\int_0^z (-c_{\text{dir}})d\zeta, \tag{S.22}$$

$$E_{\text{dif}}(z) = c^+ e^{-k^+ z} + xE_{\text{dir}}(z), \tag{S.23}$$

$$E_u(z) = c^+ r^+ e^{-k^+ z} + yE_{\text{dir}}(z). \tag{S.24}$$

S4 Miniziation algorithm

In this section we provide information about the procedure used to minimize the functional J and determine the optimal parameters. The minimzation algorithm is based on the computation on the computation of the Cost function that can be done analytically in terms of the IOPs (Chl-a, NAP, CDOM) and on the correpsonding Jacobian that facilitate the algorithm in finding minima. For each day of the time series considered, a separate inversion is performed with the following initial guess values for the IOPs Chl-a=0.4 $mg\ Chl\ m^{-3}$, CDOM=0.5 $mg\ C\ m^{-3}$, NAP=10. $mg\ C\ m^{-3}$.

The number of iteration for algorithm convergence depends on the period of the year, in fact the initial solution can be more or less "near" the optimized one. The stopping condition is based on the gradient (the treshold is gradient smaller than 10^-6).

p

S5 Analysis for NAP backscattering from avaialble data at BOOUSSOLE

To analyze better this discrepancy as first instance we analyzed all the data for the period 2005-2012 available for BOUSSOLE. We considered the seasonal variability of the spectra of bb_p reported in figure fig.S5. The goal is to understand if our modelling assumptions, spectral dependence of scattering are compatible in term of shape and magnitude to the observed data. In our modelling hypotheses two constituents concur to scattering, phytoplankton and NAP. The scattering spectra shown in fig.S5 have higher values at 442 nm and then lower values for 488 nm, in some cases (e.g. May, Jun) increasing again at 555nm. We start considering NAP with a spectral dependence $f(\lambda)$ expressed as a power law $f(\lambda) = (555/\lambda)^\eta$, and consider all the possible constituents examined in[15], see their Table 2, with the corresponding $bbp(442)/bbp(488)$

- Small Organic Detritus ($\eta = 0.5$) $\rightarrow bbp(442)/bbp(488) = 1.05$
- Large Organic Detritus ($\eta = 0$) $\rightarrow bbp(442)/bbp(488) = 1$
- Small Minerals ($\eta = 0.97$) $\rightarrow bbp(442)/bbp(488) = 1.1$
- Large Minerals ($\eta = 0.22$) $\rightarrow bbp(442)/bbp(488) = 1.02$

The evaluation of the ratio of back-scattering between wavelengths (i.e. $bbp(442)/bbp(488)$) indicates that under these model hypotheses scattering by NAP from the 4 considered compounds should be flatter with respect to what shown in fig.S5, in fact using the adopted formula we get a maximum slope 10% for the case of Small Minerals. In terms of back-scattering magnitude we could use the available data of nap absorption. In this case the average NAP absorption for the BOUSSOLE data at the reference wavelenght (440 nm) is 0.007 m^{-1} considering the average values for the months June, July, August in the years 2003-2012. Using the formulas in[15] used also in the present study:

$$a^*(\lambda) = c_1 + c_2 \exp^{-s(\lambda-440)}$$
$$b_p^*(\lambda) = b^*(555)(\frac{555}{\lambda})^\eta$$
$$b_{bp}^*(\lambda) = \tilde{b}_{bp} b_p^*(\lambda)$$

we can derive the back-scattering at Boussole at different wavelengths noting that as shown before the spectra for any of the 4 constituents is flat, values are lower for the organic compounds but higher for the inorganic compounds. If we consider the role of phytoplankton, the average chlorophyll value during summer period, as defined above, is 0.17 $mgchl\ m^{-3}$, this value needs to be converted in carbon and using the formulation for $\theta = 0.005$ considering maximal PAR exposition we get $34 mgC\ m^{-3}$. Therefore the phytoplankton contribution to backscattering could be relevant considering the mass specific back-scattering of $2.3\ 10^{-5}\ m^2\ mgC^{-1}$ we get a back scattering by phytoplankton of $0.0018 m^{-1}$ that could explain on average the observed values. But, also in the case of phytoplankton, a marked negative slope in backscattering is required to reproduce the observed spectral shape.

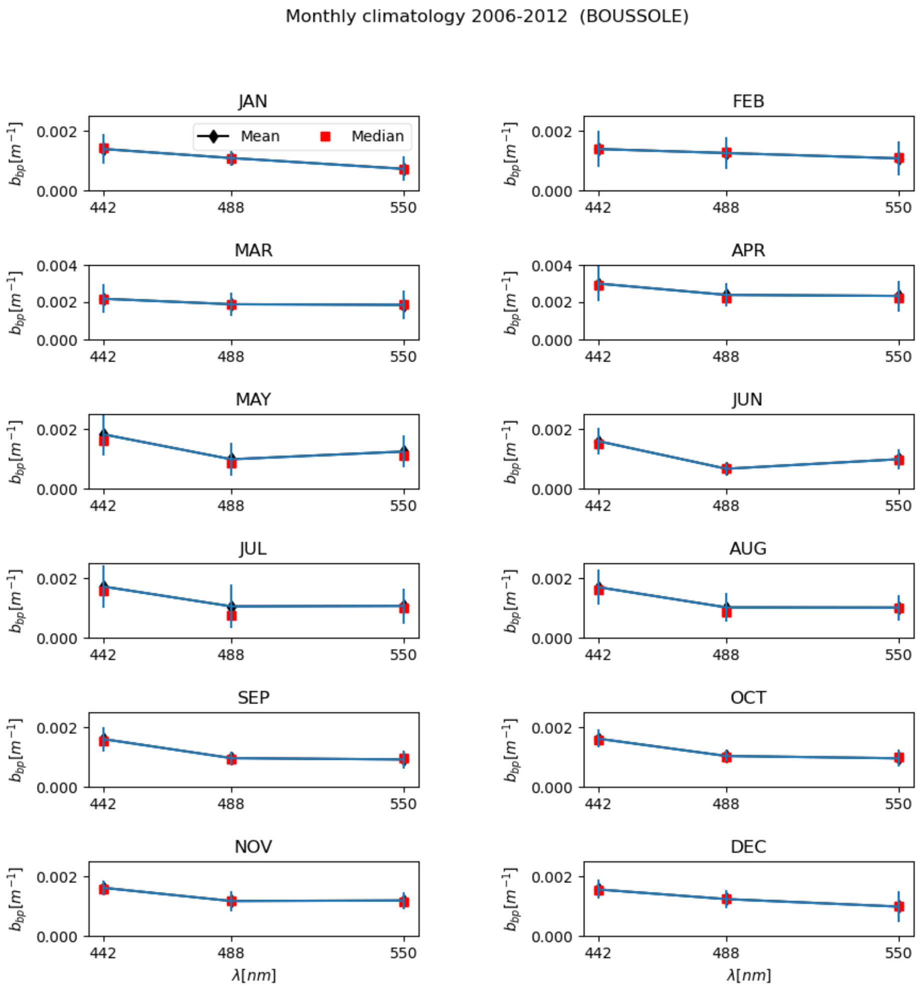

Figure S5. Monthly aggregated climatological values of b_{bp} at BOUSSOLE station.

Table S1. Estimates of b_{bp}, based on absorption data at BOUSSOLE using the formulas derived in Gallegos et al. 2011, values referes to summer period.

λ	Small Organic Detritus $b_{bp}[m^{-1}]$	Large Organic Detritus $b_{bp}[m^{-1}]$	Small Minerals $b_{bp}[m^{-1}]$	Large Minerals $b_{bp}[m^{-1}]$
412	0.000637	0.000312	0.004658	0.001548
442	0.000615	0.000312	0.004351	0.001524
488	0.000584	0.000312	0.003937	0.001490
510	0.000572	0.000312	0.003787	0.001477
555	0.000549	0.000312	0.003489	0.001450

S6 Sensitivity analysis results

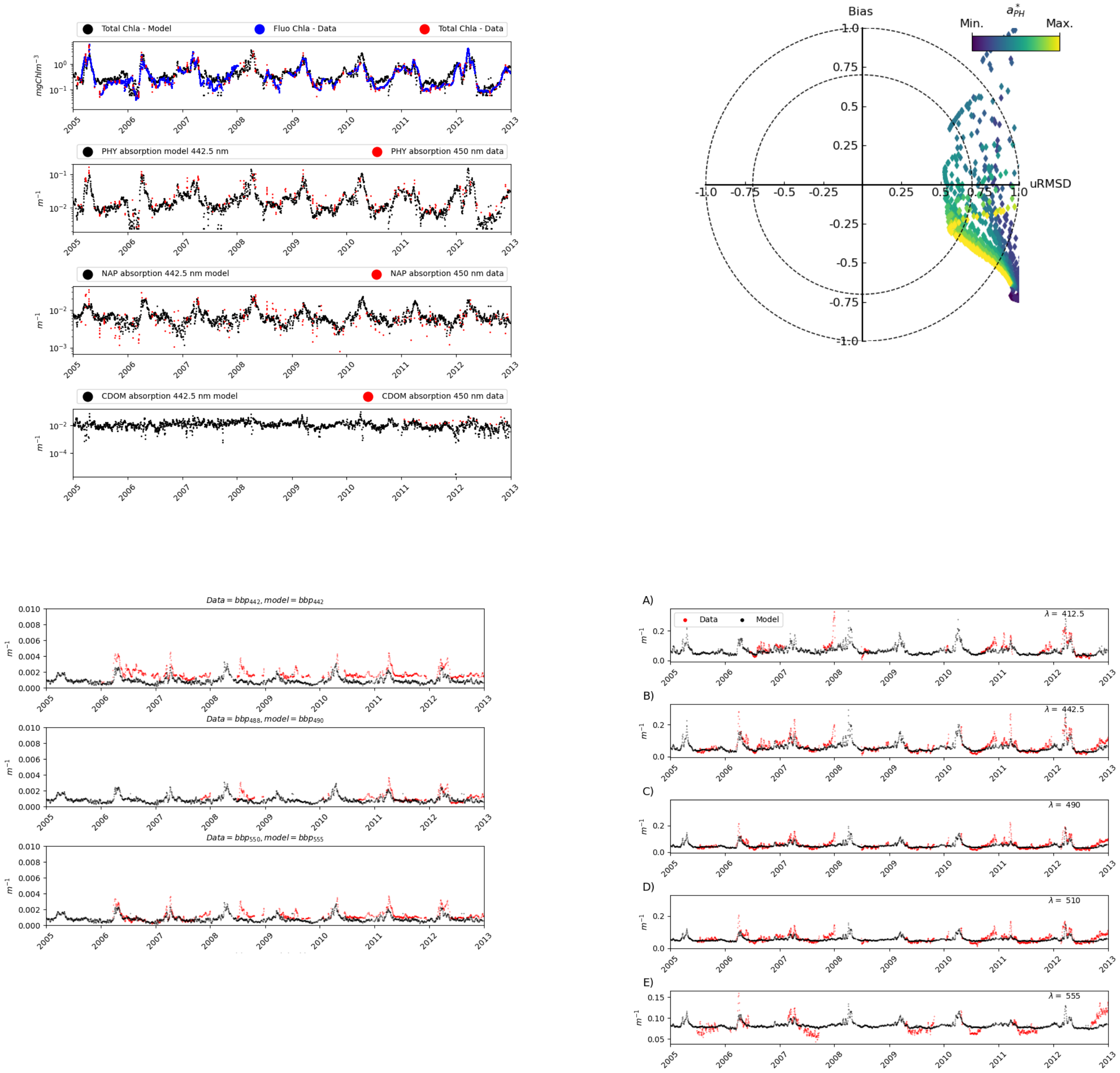

Figure S6. Best skill configuration for $Chl-a$for the sensitivity experiment performed in EXP-1. The diagrams are the same as in the main text.

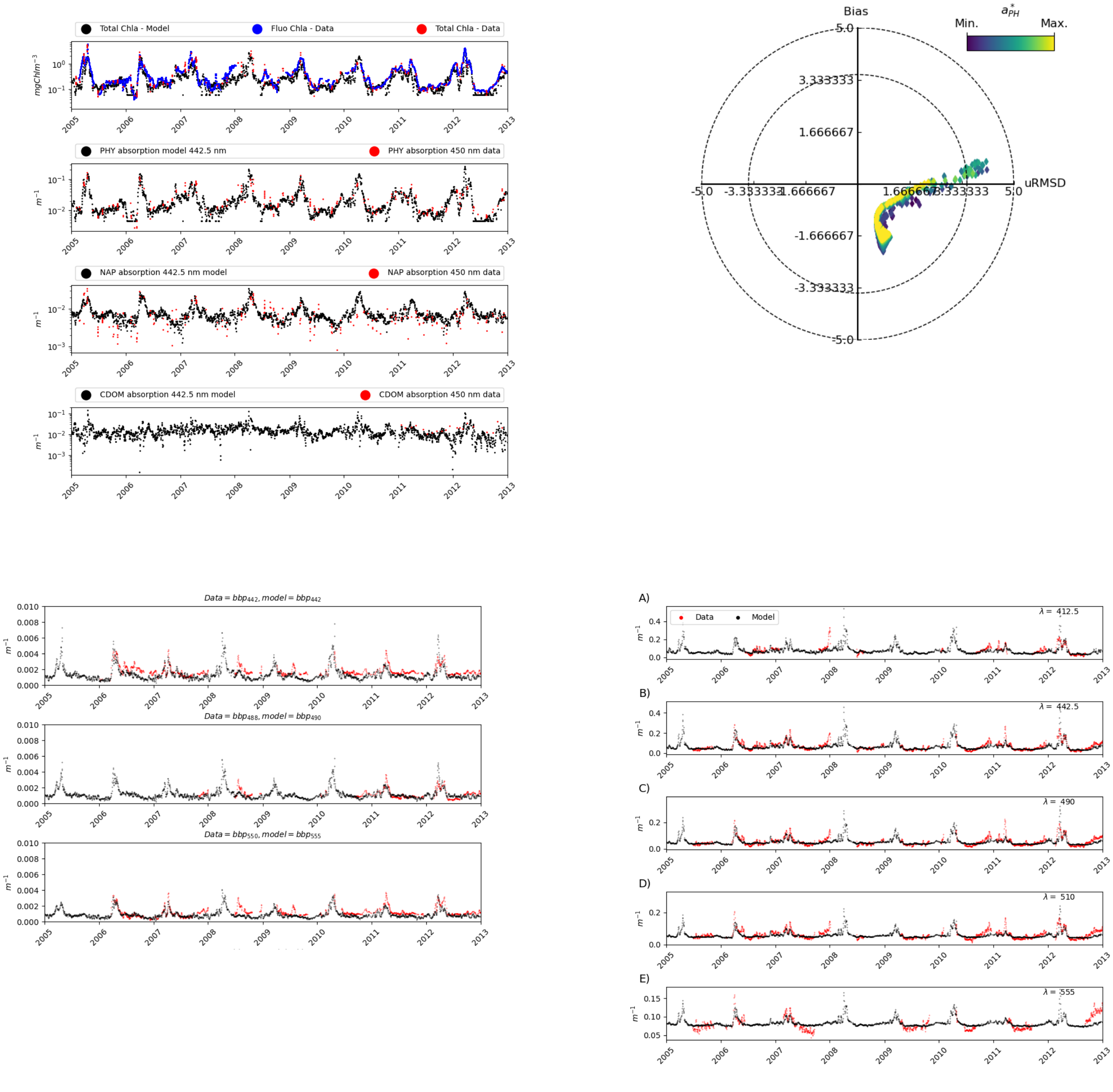

Figure S7. Best skill configuration for $bb_{442}$for the sensitivity experiment performed in EXP-1. The diagrams are the same as in the main text.

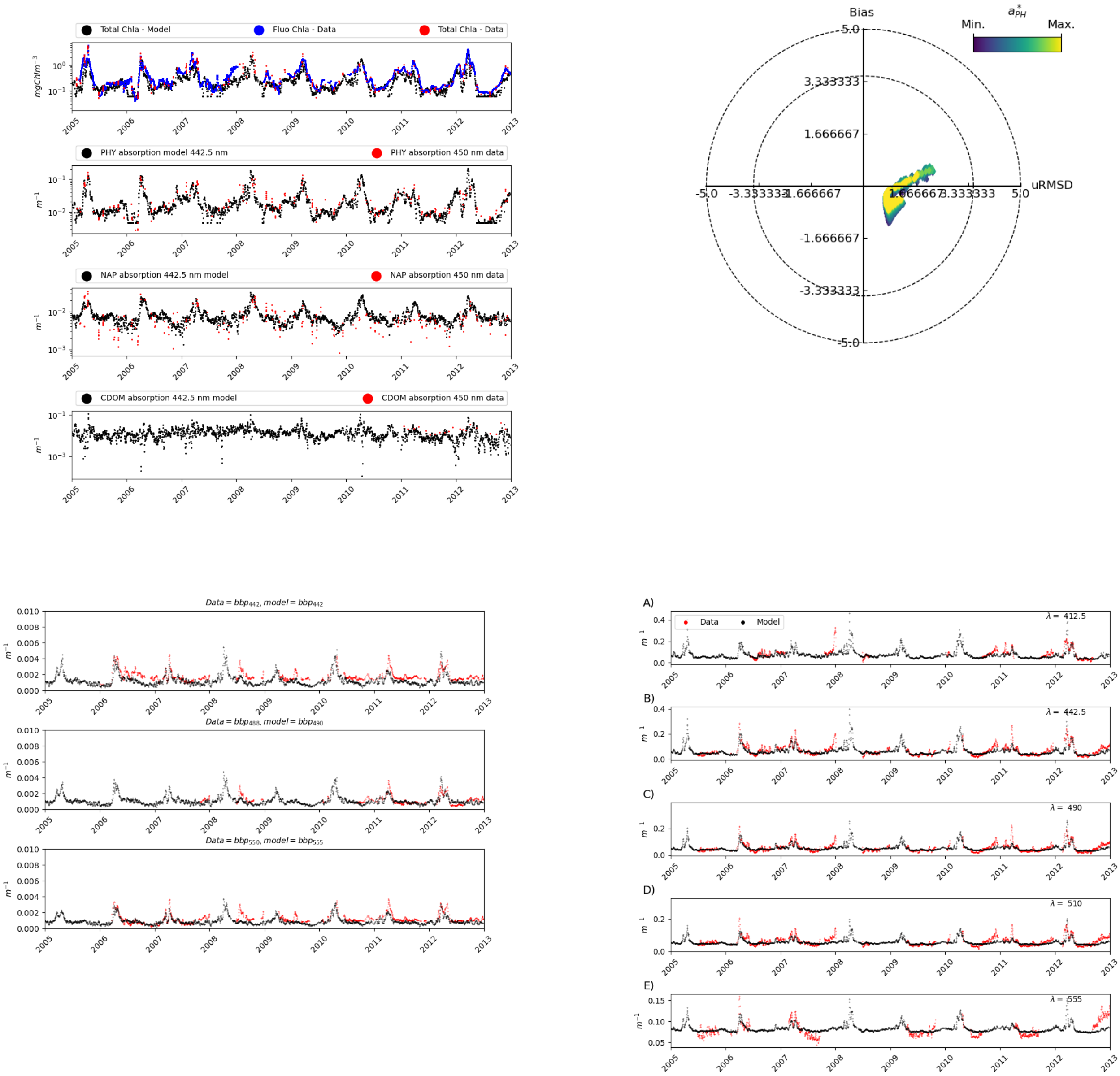

Figure S8. Best skill configuration for $bb_{490}$for the sensitivity experiment performed in EXP-1. The diagrams are the same as in the main text.

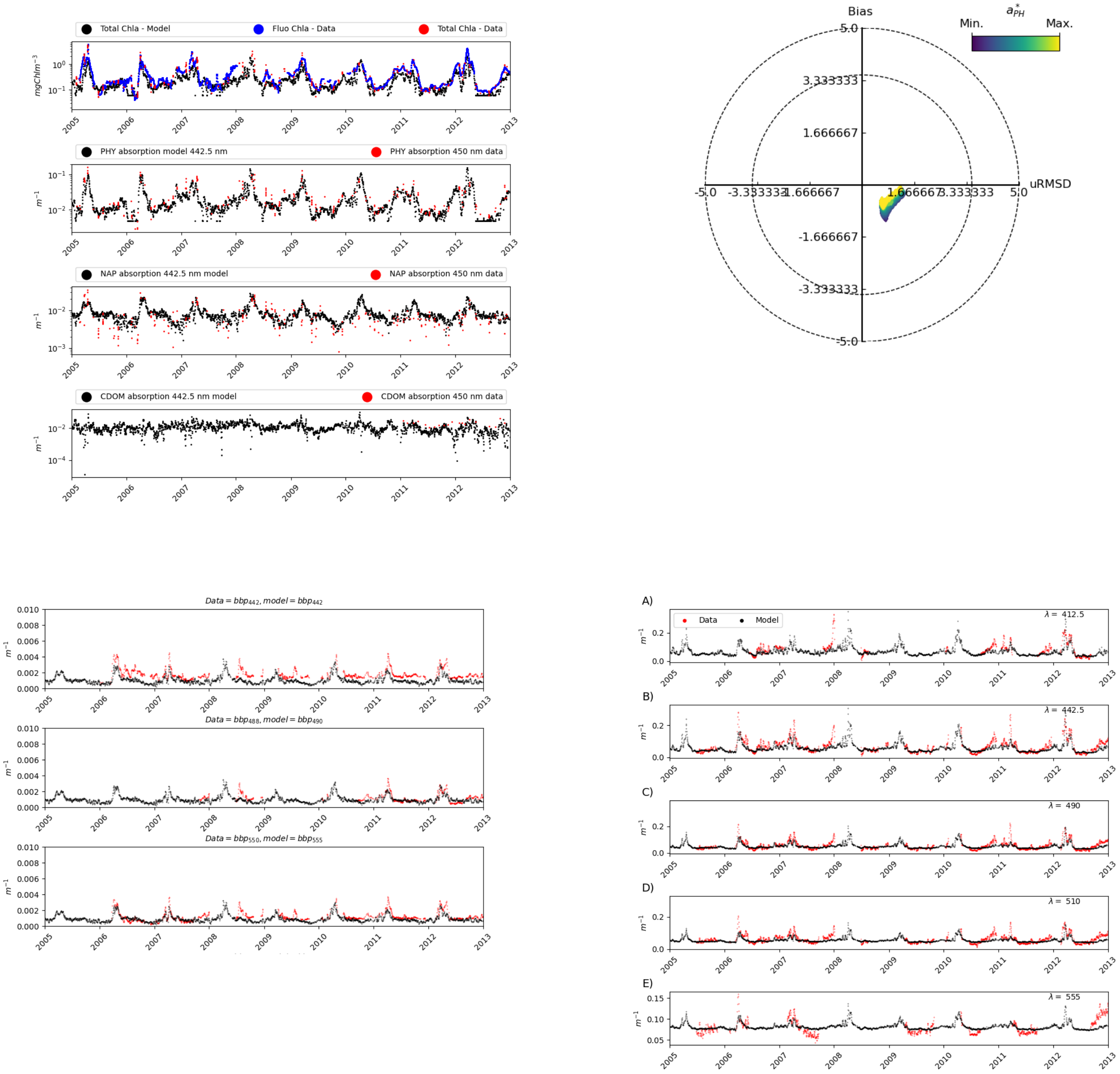

Figure S9. Best skill configuration for $bb_{555}$for the sensitivity experiment performed in EXP-1. The diagrams are the same as in the main text.

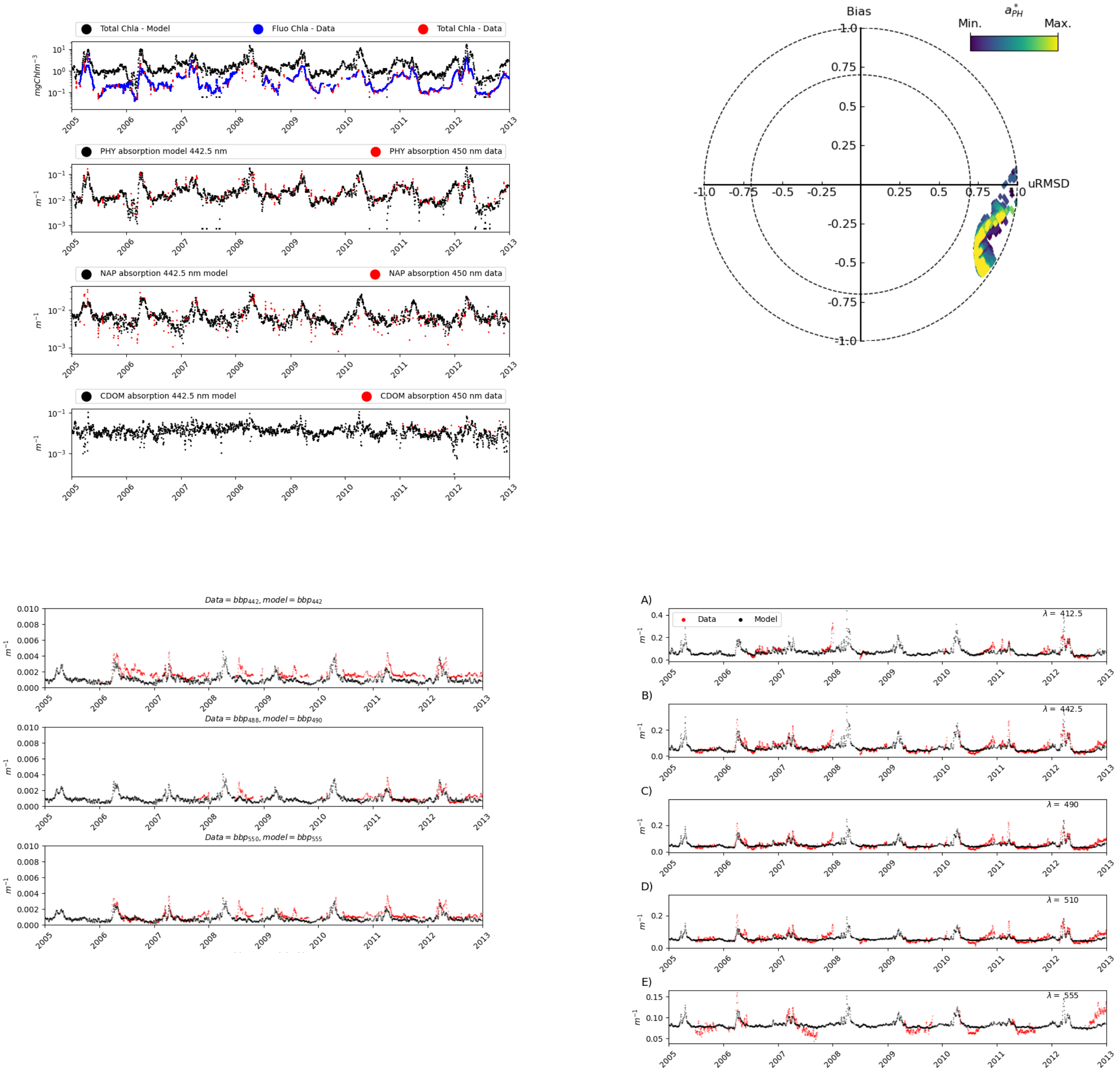

Figure S10. Best skill configuration for $kd_{442.5}$for the sensitivity experiment performed in EXP-1. The diagrams are the same as in the main text.

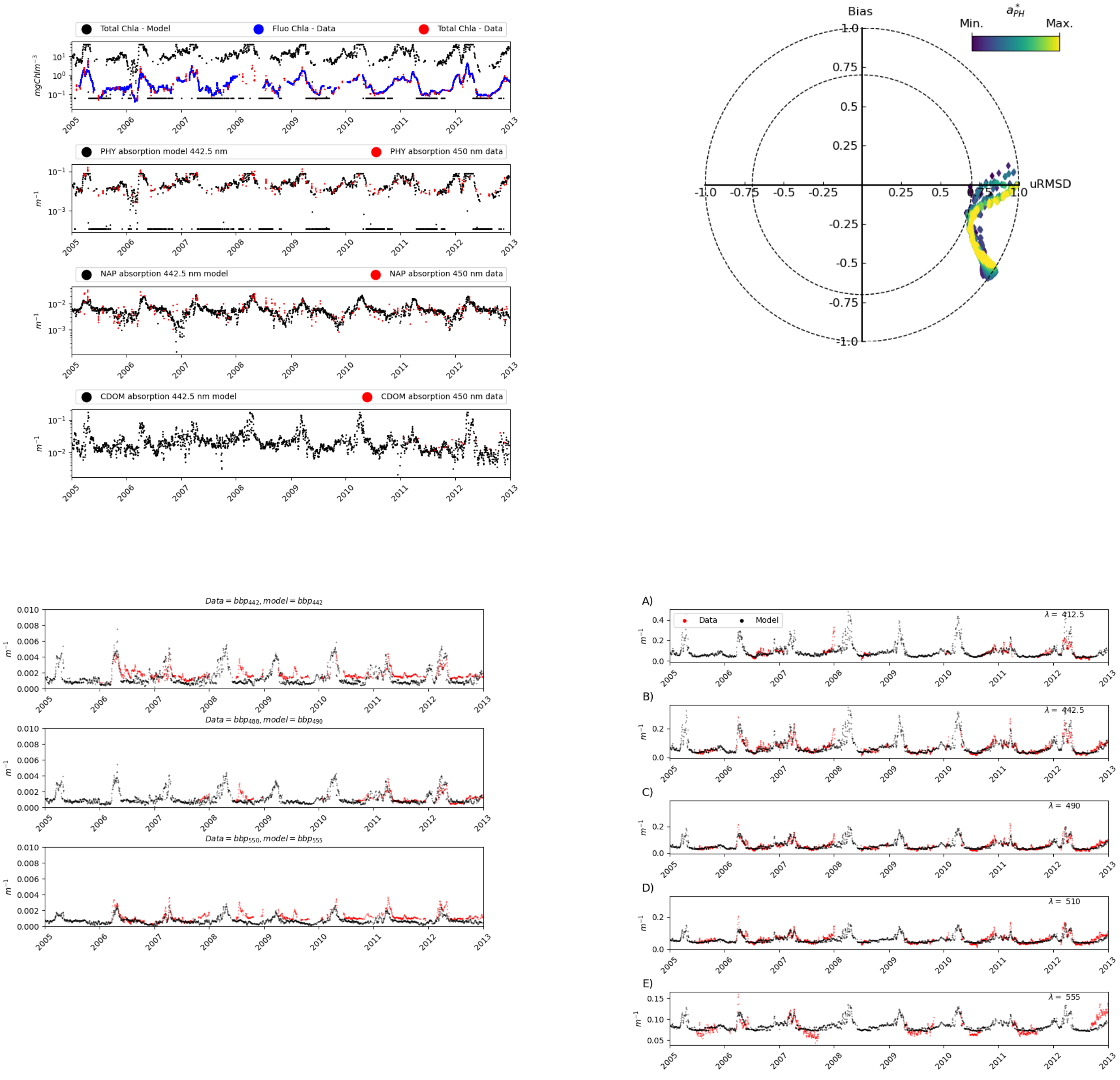

Figure S11. Best skill configuration for $kd_{510}$for the sensitivity experiment performed in EXP-1. The diagrams are the same as in the main text.

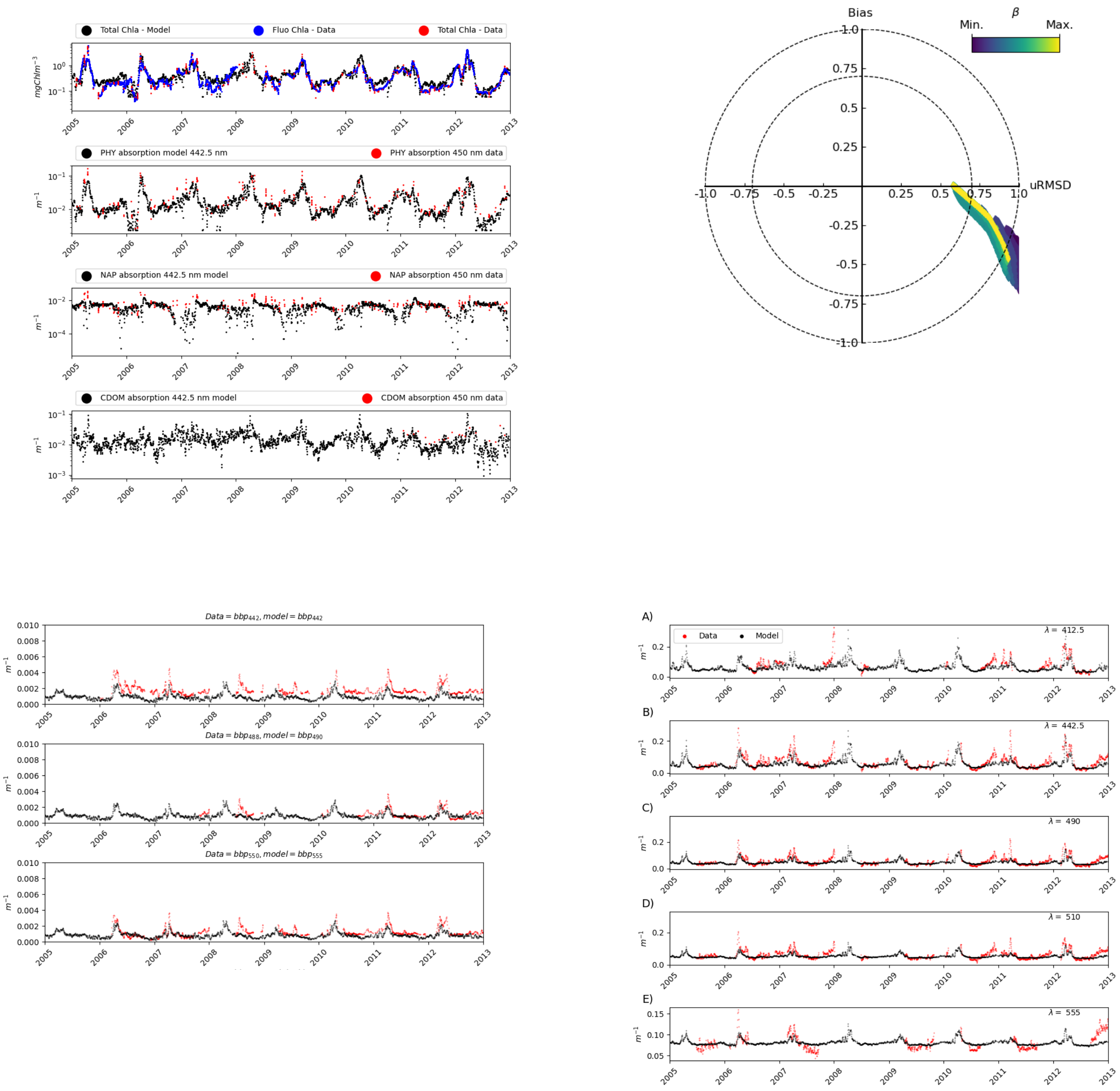

Figure S12. Best skill configuration for $Chl-a$for the sensitivity experiment performed in EXP-2. The diagrams are the same as in the main text.

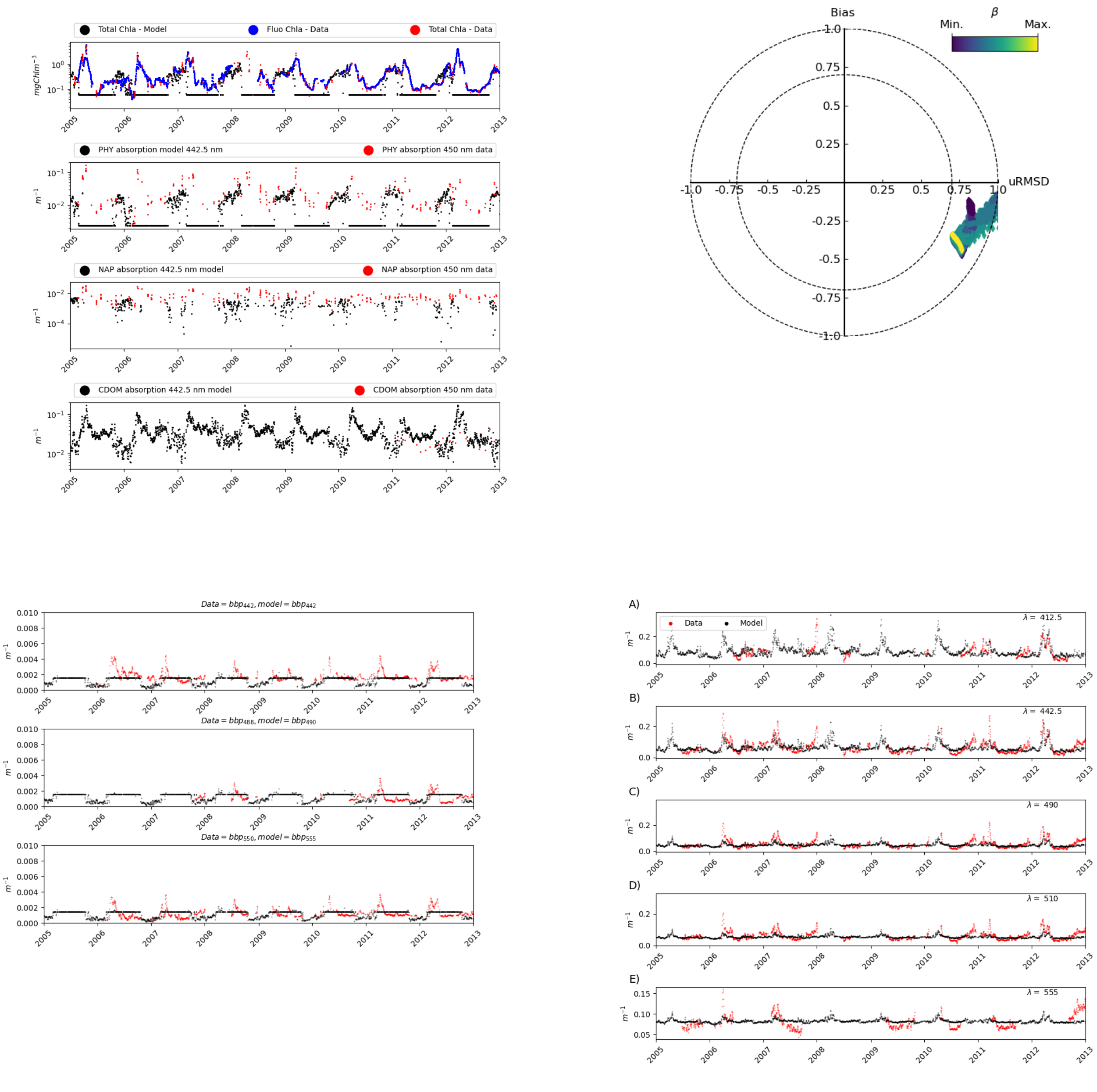

Figure S13. Best skill configuration for $bb_{442}$for the sensitivity experiment performed in EXP-2. The diagrams are the same as in the main text.

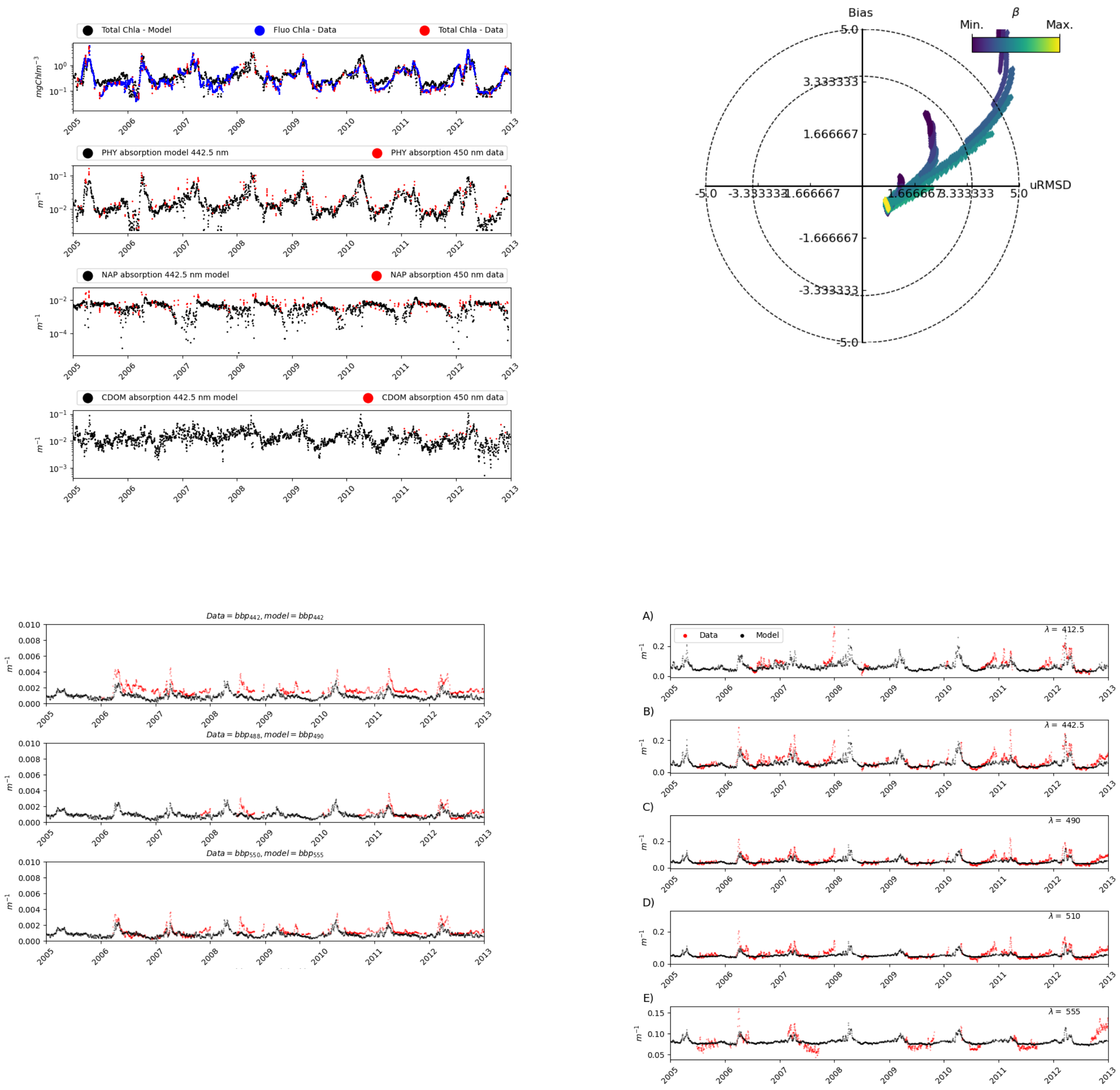

Figure S14. Best skill configuration for $bb_{490}$for the sensitivity experiment performed in EXP-2. The diagrams are the same as in the main text.

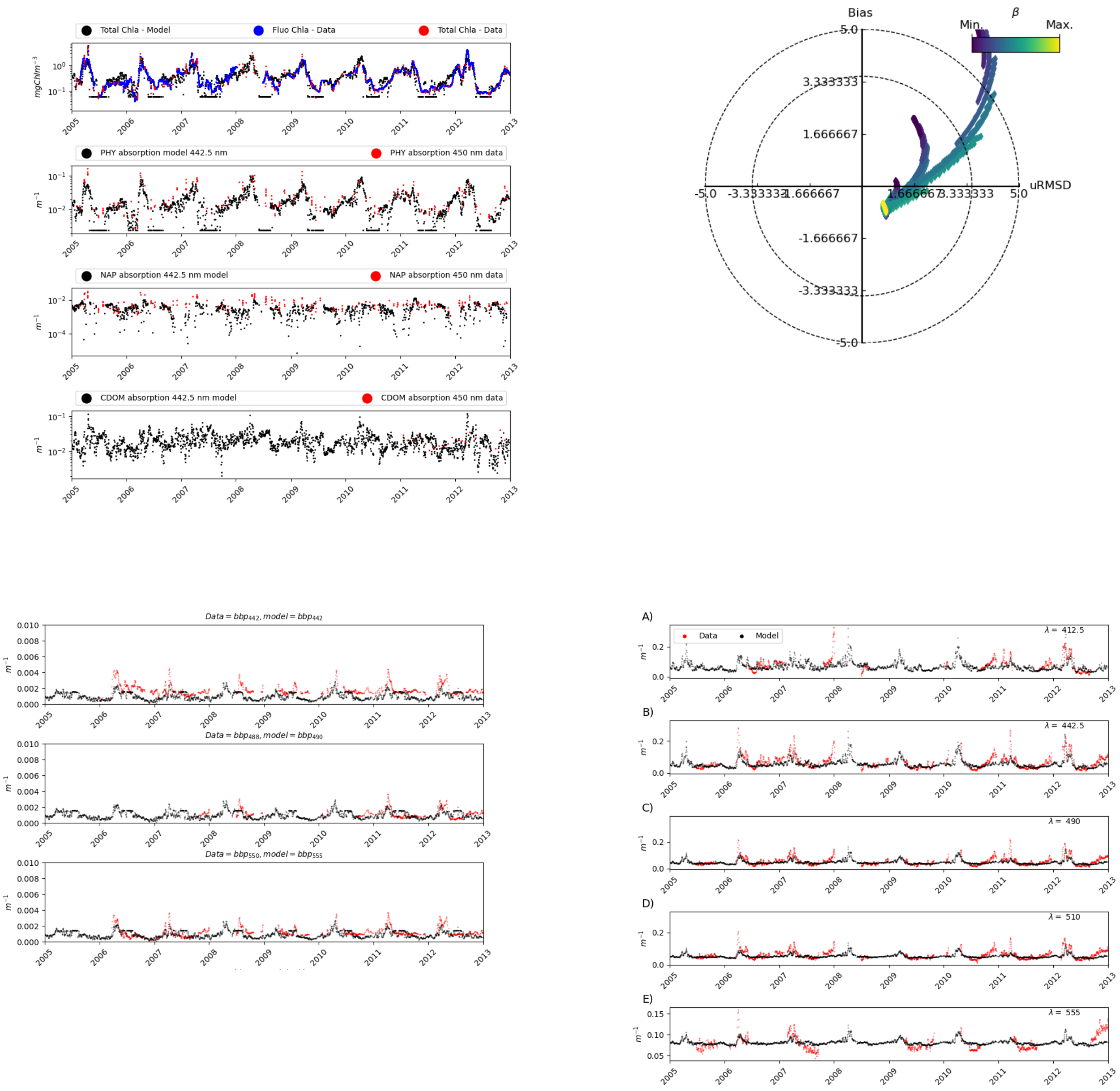

Figure S15. Best skill configuration for $bb_{555}$for the sensitivity experiment performed in EXP-2. The diagrams are the same as in the main text.

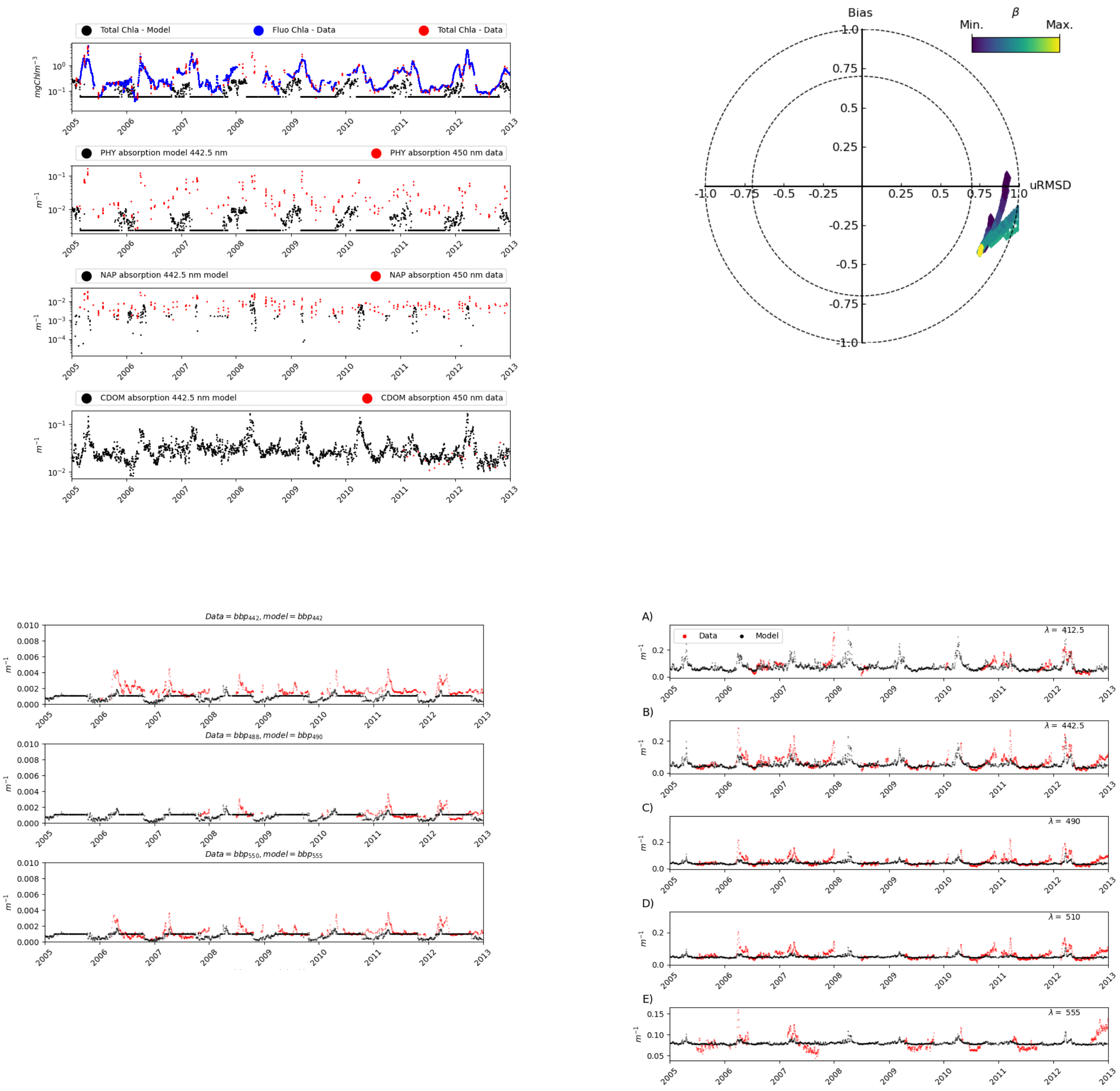

Figure S16. Best skill configuration for $Kd_{412.5}$for the sensitivity experiment performed in EXP-2. The diagrams are the same as in the main text.

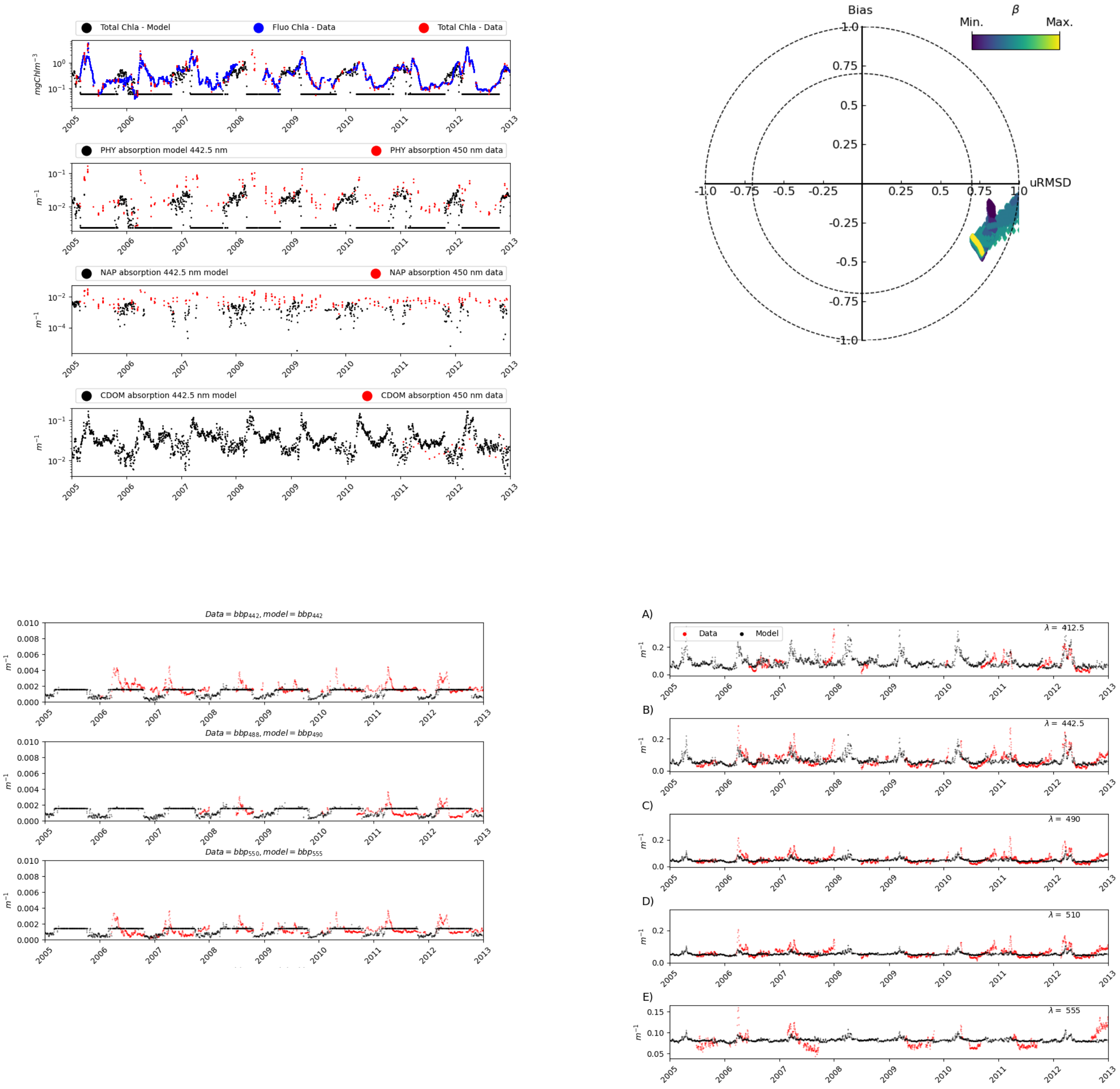

Figure S17. Best skill configuration for $kd_{442.5}$for the sensitivity experiment performed in EXP-2. The diagrams are the same as in the main text.

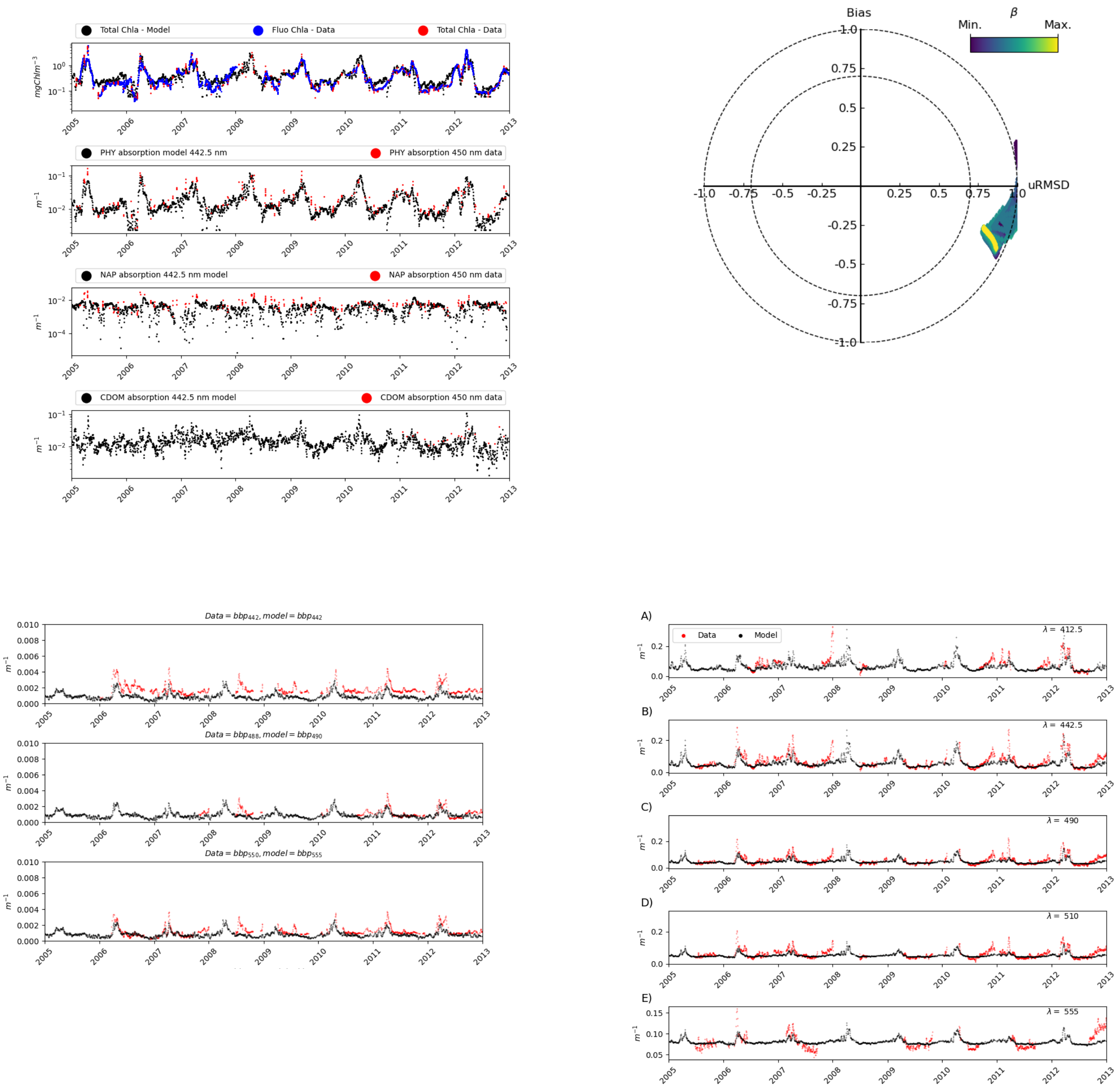

Figure S18. Best skill configuration for $kd_{490}$for the sensitivity experiment performed in EXP-2. The diagrams are the same as in the main text.

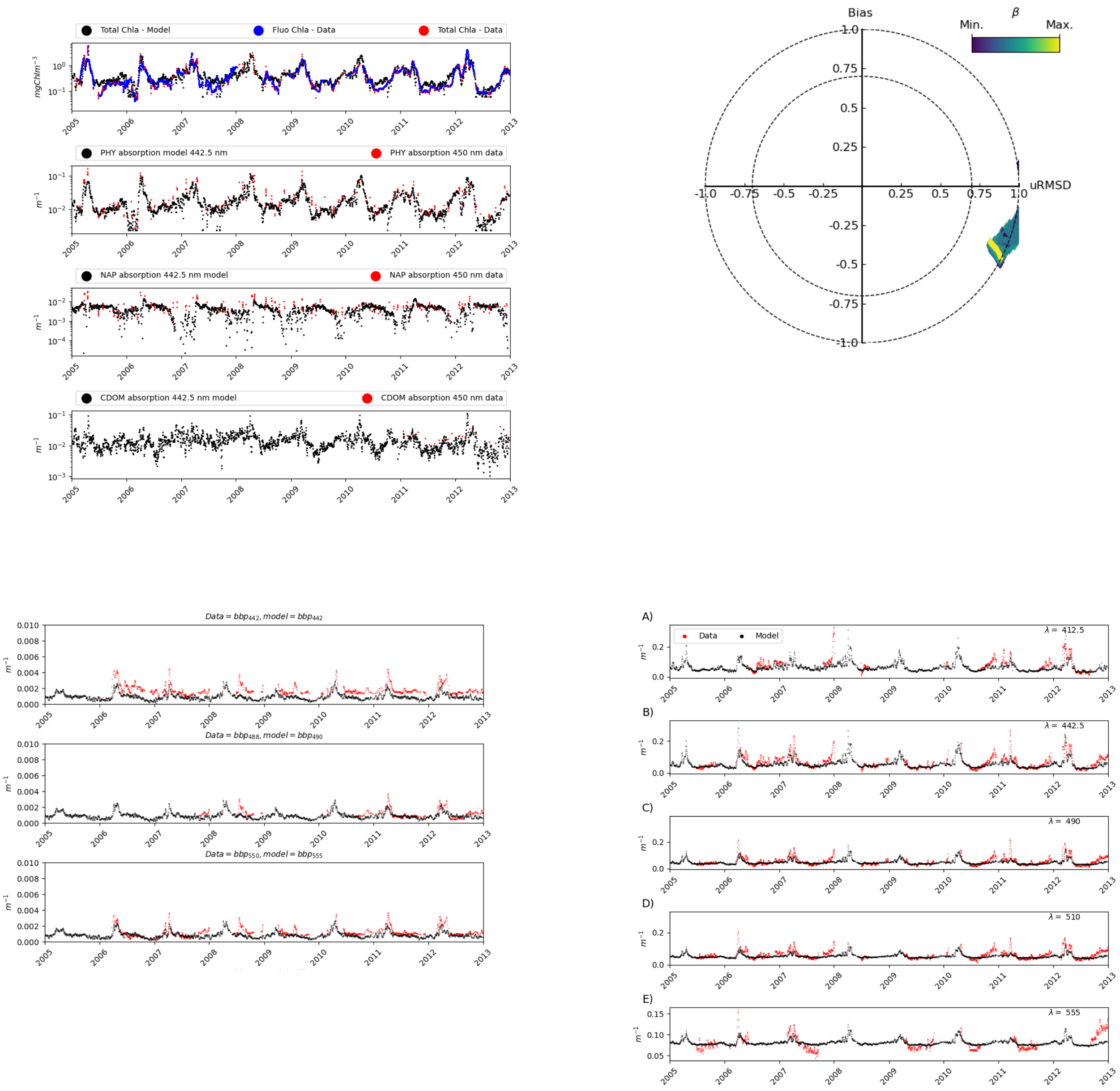

Figure S19. Best skill configuration for $kd_{510}$for the sensitivity experiment performed in EXP-2. The diagrams are the same as in the main text.

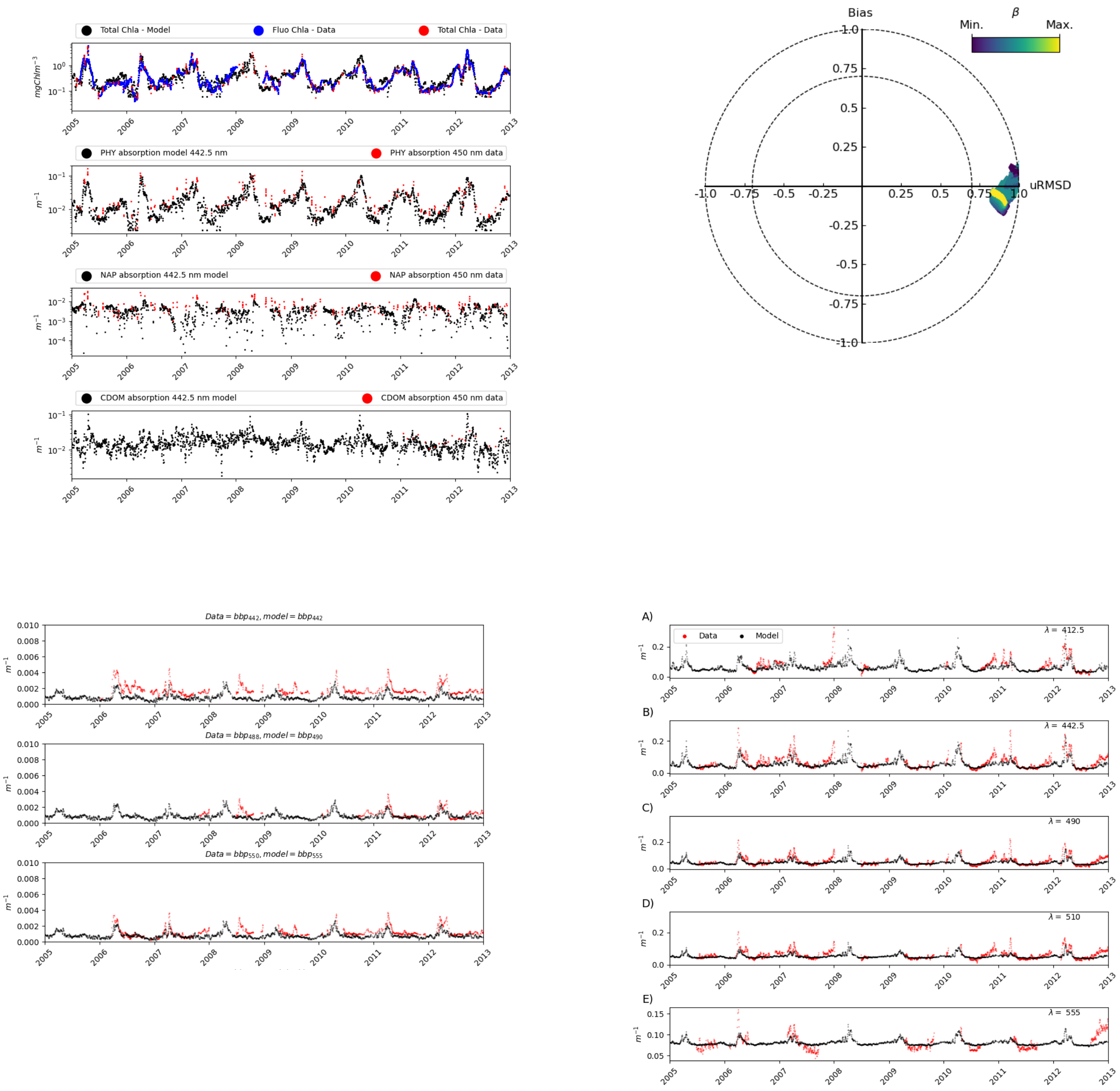

Figure S20. Best skill configuration for $kd_{555}$for the sensitivity experiment performed in EXP-2. The diagrams are the same as in the main text.

S7 Photoprotective pigments climatology at BOUSSOLE

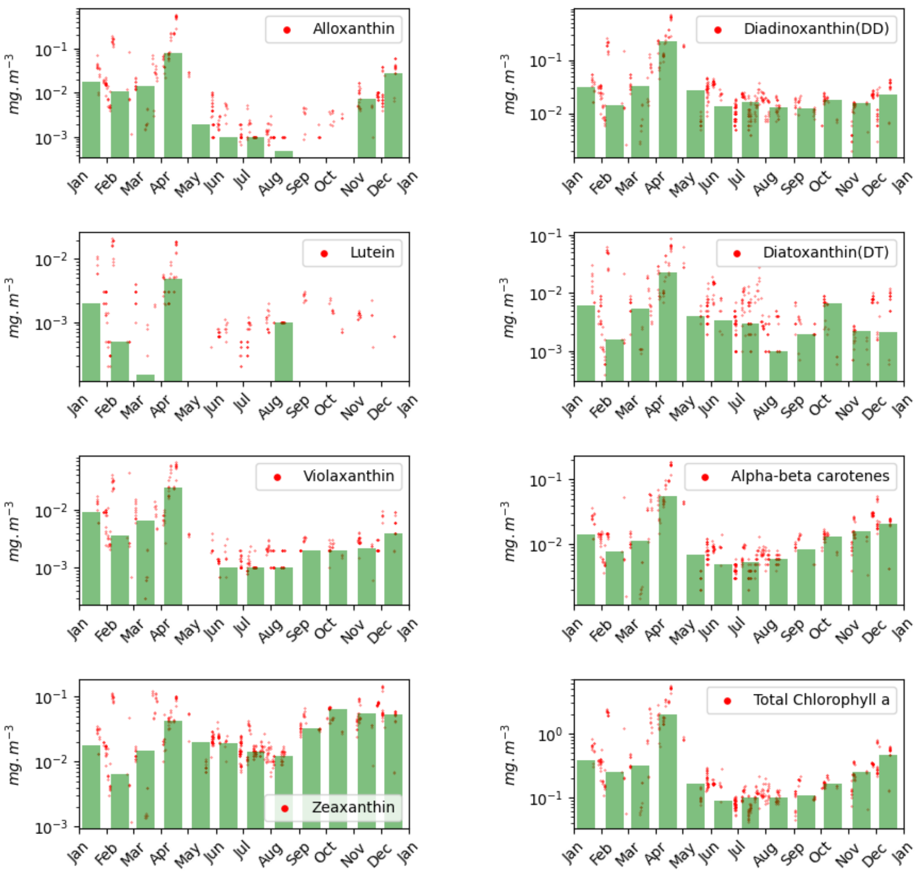

Figure S21. Reconstruction of pigments at the BOUSSOLE site. Data cover the period 2001 to 2006 and are monthly aggregated. Green bar are median values.

References

1. Met Office. *Cartopy: a cartographic python library with a Matplotlib interface*. Exeter, Devon (2010 - 2015).
2. Gregg, W. W. & Rousseaux, C. S. Simulating PACE Global Ocean Radiances. *Front. Mar. Sci.* **4**, DOI: 10.3389/fmars.2017.00060 (2017).
3. Dutkiewicz, S. *et al.* Capturing optically important constituents and properties in a marine biogeochemical and ecosystem model. *Biogeosciences* **12**, 4447–4481, DOI: 10.5194/bg-12-4447-2015 (2015).
4. Lazzari, P. *et al.* CDOM Spatiotemporal Variability in the Mediterranean Sea: A Modelling Study. *J. Mar. Sci. Eng.* **9**, 176, DOI: 10.3390/jmse9020176 (2021).
5. Aas, E. & Højerslev, N. K. Analysis of underwater radiance observations: Apparent optical properties and analytic functions describing the angular radiance distribution. *J. Geophys. Res. Ocean.* **104**, 8015–8024, DOI: 10.1029/1998JC900088 (1999).
6. Lee, Z., Carder, K. L. & Arnone, R. A. Deriving inherent optical properties from water color: a multiband quasi-analytical algorithm for optically deep waters. *Appl. Opt.* **41**, 5755, DOI: 10.1364/AO.41.005755 (2002).
7. Lee, Z. *et al.* Penetration of UV-visible solar radiation in the global oceans: Insights from ocean color remote sensing: PENETRATION OF UV-VISIBLE SOLAR LIGHT. *J. Geophys. Res. Ocean.* **118**, 4241–4255, DOI: 10.1002/jgrc.20308 (2013).
8. Cloern, J. E., Grenz, C. & Vidergar-Lucas, L. An empirical model of the phytoplankton chlorophyll : carbon ratio-the conversion factor between productivity and growth rate. *Limnol. Oceanogr.* **40**, 1313–1321, DOI: 10.4319/lo.1995.40.7.1313 (1995).
9. Lazzari, P. *et al.* Assessment of the spectral downward irradiance at the surface of the mediterranean sea using the radiative ocean-atmosphere spectral irradiance model (OASIM). *Ocean. Sci.* **17**, 675–697, DOI: 10.5194/os-17-675-2021 (2021).
10. Geider, R., MacIntyre, H. & Kana, T. Dynamic model of phytoplankton growth and acclimation:responses of the balanced growth rate and the chlorophyll a:carbon ratio to light, nutrient-limitation and temperature. *Mar. Ecol. Prog. Ser.* **148**, 187–200, DOI: 10.3354/meps148187 (1997).
11. Ronald, J. & Zaneveld, V. Remotely sensed reflectance and its dependence on vertical structure: a theoretical derivation. *Appl. Opt.* **21**, 4146, DOI: 10.1364/AO.21.004146 (1982).
12. Byrd, R. H., Lu, P., Nocedal, J. & Zhu, C. A Limited Memory Algorithm for Bound Constrained Optimization. *SIAM J. on Sci. Comput.* **16**, 1190–1208, DOI: 10.1137/0916069 (1995).
13. Zhu, C., Byrd, R. H., Lu, P. & Nocedal, J. Algorithm 778: L-BFGS-B: Fortran subroutines for large-scale bound-constrained optimization. *ACM Transactions on Math. Softw.* **23**, 550–560, DOI: 10.1145/279232.279236 (1997).
14. Virtanen, P. *et al.* SciPy 1.0: Fundamental Algorithms for Scientific Computing in Python. *Nat. Methods* **17**, 261–272, DOI: 10.1038/s41592-019-0686-2 (2020).
15. Gallegos, C. L., Werdell, P. J. & McClain, C. R. Long-term changes in light scattering in Chesapeake Bay inferred from Secchi depth, light attenuation, and remote sensing measurements. *J. Geophys. Res. Ocean.* **116**, 2011JC007160, DOI: 10.1029/2011JC007160 (2011).
